# MHC-II dynamics are maintained in HLA-DR allotypes to ensure catalyzed peptide exchange

**DOI:** 10.1038/s41589-023-01316-3

**Published:** 2023-05-04

**Authors:** Esam T. Abualrous, Sebastian Stolzenberg, Jana Sticht, Marek Wieczorek, Yvette Roske, Matthias Günther, Steffen Dähn, Benedikt B. Boesen, Marcos Martínez Calvo, Charlotte Biese, Frank Kuppler, Álvaro Medina-García, Miguel Álvaro-Benito, Thomas Höfer, Frank Noé, Christian Freund

**Affiliations:** 1 Protein Biochemistry, Institute for Chemistry and Biochemistry, Freie Universität Berlin, Berlin, Germany; 2 Department of Mathematics and Computer Science, Freie Universität Berlin, Berlin, Germany; 3 Department of Physics, Faculty of Science, Ain Shams University, Cairo, Egypt; 4 Core Facility BioSupraMol, Institute for Chemistry and Biochemistry, Freie Universität Berlin, Berlin, Germany; 5 Max-Delbrück-Center for Molecular Medicine, Berlin, Germany; 6 Theoretische Systembiologie (B086), Deutsches Krebsforschungszentrum, Heidelberg, Germany; 7 Microsoft Research AI4Science, Berlin, Germany; 8 Department of Physics, Freie Universität Berlin, Berlin, Germany; 9 Department of Chemistry, Rice University, Houston, TX USA

**Keywords:** Enzyme mechanisms, Peptides, Immunology, NMR spectroscopy

## Abstract

Presentation of antigenic peptides by major histocompatibility complex class II (MHC-II) proteins determines T helper cell reactivity. The MHC-II genetic locus displays a large degree of allelic polymorphism influencing the peptide repertoire presented by the resulting MHC-II protein allotypes. During antigen processing, the human leukocyte antigen (HLA) molecule HLA-DM (DM) encounters these distinct allotypes and catalyzes exchange of the placeholder peptide CLIP by exploiting dynamic features of MHC-II. Here, we investigate 12 highly abundant CLIP-bound HLA-DRB1 allotypes and correlate dynamics to catalysis by DM. Despite large differences in thermodynamic stability, peptide exchange rates fall into a target range that maintains DM responsiveness. A DM-susceptible conformation is conserved in MHC-II molecules, and allosteric coupling between polymorphic sites affects dynamic states that influence DM catalysis. As exemplified for rheumatoid arthritis, we postulate that intrinsic dynamic features of peptide–MHC-II complexes contribute to the association of individual MHC-II allotypes with autoimmune disease.

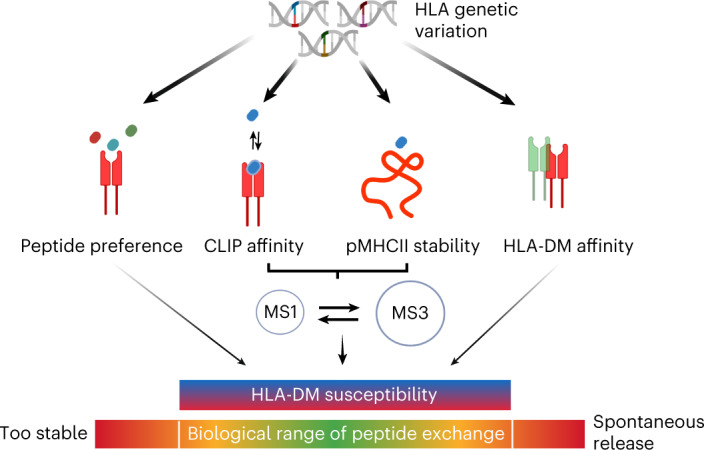

## Abstract (web summary)

By using a combined computational and experimental approach, peptide exchange of 12 abundant MHC-II allotypes is shown to maintain responsiveness to HLA-DM catalysis. HLA-DM susceptibility is postulated to contribute to disease association.

## Main

Catalyzed peptide exchange is a hallmark of antigen processing and presentation by MHC molecules^1,2^. For MHC class I (MHC-I) molecules, tapasin, as part of the larger peptide loading complex in the endoplasmatic reticulum, and TAPBPR, acting later during transport, exert the role of peptide editing^3,4^. For MHC class II (MHC-II) molecules, which typically reside in late endosomal vesicles, the noncanonical HLA molecule HLA-DM (DM) acts as peptide exchange catalyst. It engages the peptide–MHC-II complex from the site of the binding pocket that accommodates the first residue of the peptide (P1)^1,2,5–7^. Typically, DM encounters MHC-II in complex with a class II-associated invariant chain peptide (CLIP), the placeholder peptide derived from the invariant chain (CD74) that assists MHC-II proteins during folding. The MHC-II proteins HLA-DR, HLA-DQ and HLA-DP are heterodimers consisting of a highly polymorphic β-chain and an α-chain showing little (HLA-DQ and HLA-DP) or almost no (HLA-DR) polymorphism^1^. As the main DM binding site is formed by the α-chain^6^, the mode of interaction can be considered conserved over different HLA-DR (DR) protein variants (allotypes). Extensive analyses on the commonly studied HLA-DR1 (DR1) allotype (consisting of chains DRA*01:01/DRB1*01:01) have shown that peptide affinity and pocket occupancy, as for example induced by variation in the DR β-chain, tune the activity of DM^2,5,8,9^. Moreover, it was found that the intrinsic stability of peptide–MHC-II complexes correlated linearly with catalytic turnover^10^. Additionally, by analyzing different peptides with varying MHC-II binding affinities, substitutions remote from the P1 pocket were shown to influence DM susceptibility (defined as the DM-dependent enhancement of peptide dissociation rates). These findings support previous studies showing that the identity of the peptide residues occupying the P9 pocket, which is most distal to P1, has an influence on DM-catalyzed peptide exchange^8^.

It had been suggested early on that the conformational plasticity of a peptide–MHC-II (pMHCII) complex determines interaction with DM^8,11–14^. In line with that, our group had found that catalyzed peptide exchange is driven by a rare conformational state of the pMHCII complex^2,5^. This state is an intermediate that carries not only hallmarks of the crystallographically described ground state, but also features of the DM-bound structure. The most dynamic regions involve the α1 domain important for the direct interaction with DM. Interestingly, part of the β2 domain α-helix lining the P1–P4 pocket region locally unfolds, thereby defining a dynamic hot spot at the non-DM interacting site. However, whether and how natural variation at remote pockets along the binding groove affects DM interaction and susceptibility is not clear.

Therefore, we now address the question of the contribution of natural polymorphisms in the DR β-chain to catalyzed peptide exchange, while the α-chain forming the direct DM binding site remains the same. We use the universal placeholder peptide CLIP^15^ in all cases as dissociating and incoming peptide to extract the role of MHC allelic differences. We show that intrinsic stability of DR molecules does not correlate directly with DM susceptibility. Rather, the previously described occupancy of dynamic states, as it is now simulated for 12 highly abundant allotypes, explains the observed allotype-dependent DM activity. Furthermore, nuclear magnetic resonance (NMR) experiments confirm that DM binding coincides with conformational dynamics, and double mutant cycle analysis reveals allosteric coupling between polymorphic sites. Apparently, low CLIP-affinity allotypes keep uncatalyzed peptide exchange at bay, and very stable CLIP–MHC-II complexes are still susceptible to catalyzed peptide exchange.

## Results

### Biochemical description of a set of 12 DRB1 allotypes

A DR1 MHC-II protein consists of two chains, the HLA-DRA (DRA) chain and the HLA-DRB1 (DRB1) chain. Whereas the DRA chain shows a very low degree of polymorphism at the protein level (five different variants identified), the DRB1 chain is highly polymorphic, with 2,107 different protein variants identified (https://www.ebi.ac.uk/ipd/imgt/hla/about/statistics (accessed February 2022)). Here, we compare a set of MHC-II proteins consisting of the DRA1*01:01 chain and 12 different DRB1 allotypes in terms of their peptide exchange properties.

**Fig. 1:**
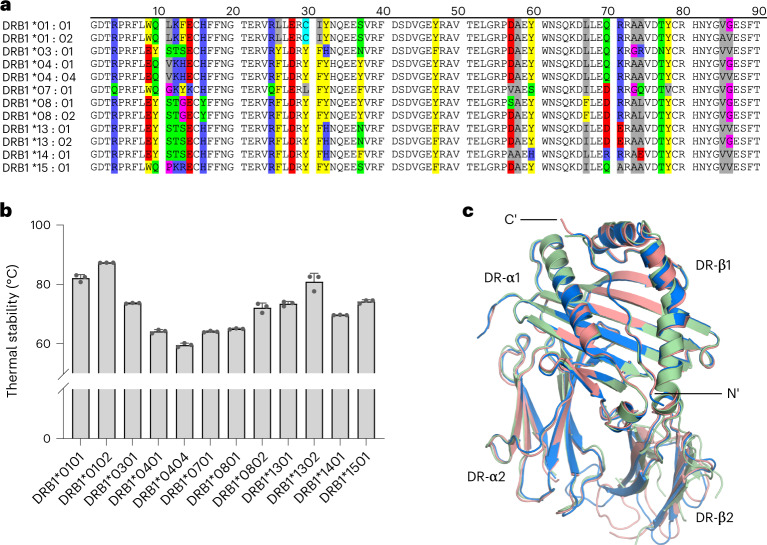
Characterization of DRB1 natural variants. **a**, Sequence alignment of the β1 domains of the investigated DRB1 natural variants. The ten most polymorphic positions (β-chain residues 10, 11, 13, 30, 37, 57, 67, 70, 71 and 74)^1^ are highlighted and colored according to their physical properties: red, polar negative (D, E); blue, polar positive (R, K, H); yellow, nonpolar aromatic (F, Y, W); green, polar neutral (S, T, Q, N); gray, nonpolar aliphatic (I, L, V, A); pink, small (G, P); cyan, Cys (C). **b**, Thermal stability of the investigated DRB1 natural variants in complex with CLIP. Data are presented as mean ± s.d. (*n* = 3 independent experiments). **c**, Structural alignment of the most thermostable allotype (DRB1*01:02 (light blue)) and two of the least thermostable allotypes (DRB1*04:01 (light red) and DRB1*07:01 (light green)).

**Extended Data Fig. 1:**
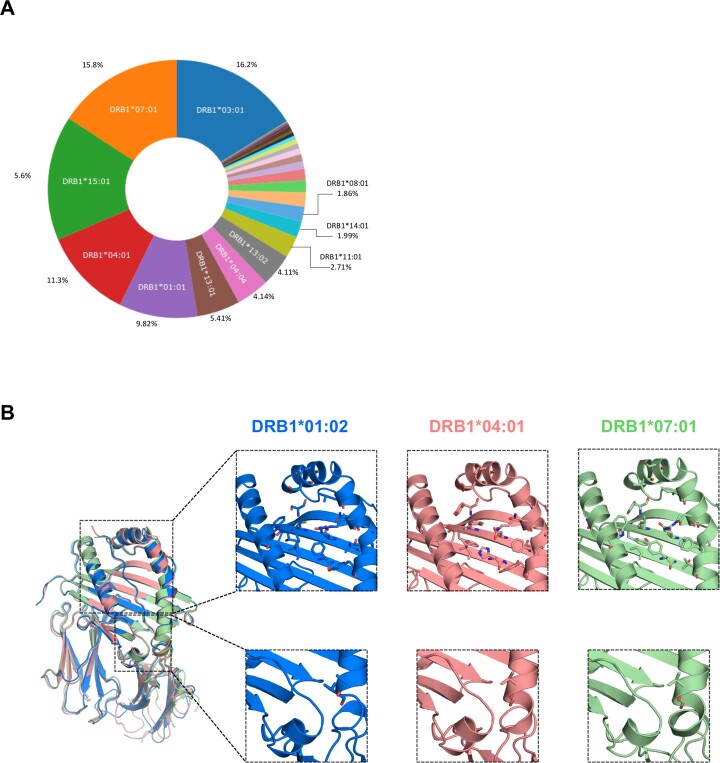
DRB1 natural variants. **a**. Shown are DRB1 allotype frequencies in the UK Biobank dataset. The pie chart was modified from the one retrieved from the Global Biobank Engine, Stanford, CA (URL: http://gbe.stanford.edu)^16^ [accessed February 2022]. The authors would like to thank the Rivas lab for making the resource available. **b**. Structures of DRB1*01:02, DRB1*04:01, and DRB1*07:01. Left: Structural alignment of DRB1*01:02 (blue), DRB1*04:01 (light red), and DRB1*07:01 (light green) Right: Magnification of the P4-P9 pocket regions (top) and P1 pocket region (bottom), with the polymorphic residues shown as sticks.

This set of proteins covers the allotypes most frequently observed in the UK Biobank population^16^ (Extended Data Fig. 1a). The investigated DRB1 proteins vary at the ten most polymorphic residues identified for DRB1^1^ (Fig. 1a). Sequence variation in the peptide binding groove alters the presented peptide repertoire, and this partially explains the observed association of some DRB1 allotypes with risk for or protection from autoimmune disease^1^. To potentially draw conclusions about the effect of individual polymorphisms, we included pairs of allotypes that differ only in few amino acids (DRB1*01:01/*01:02 (V85A and G86V), DRB1*13:01/*13:02 (V86G), DRB1*04:01/*04:04 (K71R and G86V) and DRB1*0801/*08:02 (S57D)).

The 12 DRB1 proteins were expressed in insect cells and purified preloaded with the natural placeholder peptide CLIP^5,17,18^, which preoccupies the peptide binding groove of nascent MHC-II proteins prior to peptide exchange. Next, we determined the thermal stability^19^ of the 12 purified (see Methods) CLIP–DRB1 proteins and found that it varies between 87.3 °C (DRB1*01:02) and 59.5 °C (DRB1*04:04) (Fig. 1b and Supplementary Table 1). Measurements in the presence of a large excess of CLIP peptide lead to only a small additional stabilization (<5 °C) for allotypes of both high and low thermal stability (Supplementary Table 2). This ensures that detected values are not dominated by differential loss of CLIP peptide. Our results are consistent with previously reported thermal stability values of CLIP–DRB1 complexes^5,20^.

A difference in thermal stability might result from differential CLIP affinity as well as distinct intrinsic stability of the MHC-II itself. We solved the structures of CLIP-bound DRB1*01:02, DRB1*04:01 and DRB1*07:01 (see Supplementary Table 3; Protein Data Bank (PDB) codes 7YX9, 7YXB and 7Z0Q). Although these complexes cover a range of thermal stabilities between 64.1 °C (DRB1*07:01) and 87.3 °C (DRB1*01:02), they all show essentially the same three-dimensional fold (Fig. 1c and Extended Data Fig. 1b). The three structures closely resemble the published CLIP–MHC-II crystal structures DRB1*01:01/CLIP (PDB code 3PDO (ref. ^21^)) and DRB1*03:01/CLIP (PDB code 1A6A (ref. ^22^)), as shown by Cα root mean square deviation (RMSD) values below 1 Å (Supplementary Table 4). Thus, these CLIP-bound allotypes apparently occupy the stable ground-state structure with CLIP bound in the same canonical orientation and register.

Although we see no significant differences in ground-state structure, we still expect polymorphisms to influence CLIP affinity and exchange, as they may alter the dynamic properties of the peptide binding groove.

### Polymorphisms in DRB1 affect intrinsic pMHCII stability

Polymorphisms in the MHC-II β-chain are located in the peptide binding groove and influence the affinity toward different peptides^1^. Assuming a very simple model, in which solely CLIP affinity determines the intrinsic CLIP off-rate and thereby the thermal stability of a pMHCII complex, these parameters should be correlated as measures of intrinsic stability. In line with that, it has previously been observed that single point mutations in DRB1 that break H-bonds to the peptide go along with reduced thermal stability^5^.

**Extended Data Fig. 2:**
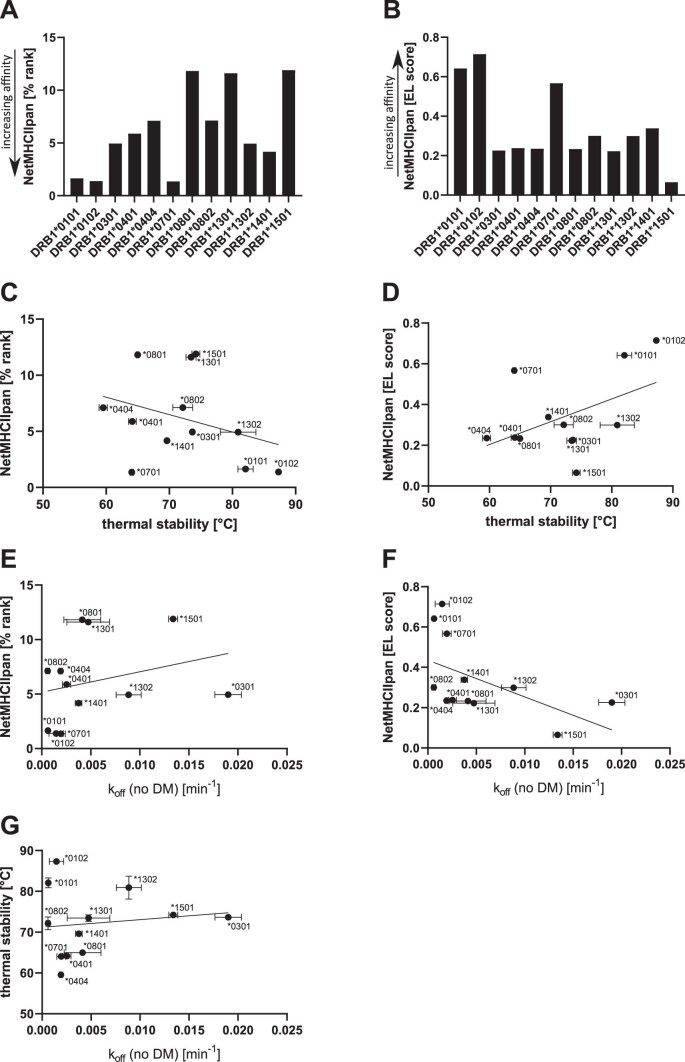
Parameter of intrinsic CLIP–MHC-II stability. Plotted are CLIP binding scores (%rank in **a** and EL score in **b**) as predicted by NetMHCIIPan^23,24^ for the twelve DRB1 allotypes. Correlation plots are shown for NetMHCIIpan %rank (**c**) or EL score (**d**) versus thermal stability (R^2^ = 0.11 (**C**) and R^2^ = 0.23 (**D**)). Correlation plots are shown for NetMHCIIpan %rank (**e**) or EL score (**f**) versus intrinsic CLIP off-rate (R^2^ = 0.07 (**E**) and R^2^ = 0.28 (**F**)), as well as for thermal stability versus intrinsic CLIP-off rate (**g**, R^2^ = 0.02). Data for thermal stability and intrinsic off-rate are presented as mean values ± SD (n = 3 independent experiments).

To challenge the validity of such a simple model, we predicted a CLIP peptide binding score for every individual allotype using NetMHCIIpan^23,24^ (see Methods). For all analyzed allotypes, CLIP was predicted to bind with its canonical core motif (MRMATPLLM). NetMHCIIpan output values varied by a factor of ten between allotypes (Extended Data Fig. 2a, b and Supplementary Table 1). Under the assumption that CLIP affinity predominantly defines CLIP–MHC-II thermal stability, a correlation should be observed. Interestingly, there is no linear correlation between predicted CLIP affinity and thermal stability over the 12 different allotypes (Extended Data Fig. 2c, d).

**Fig. 2:**
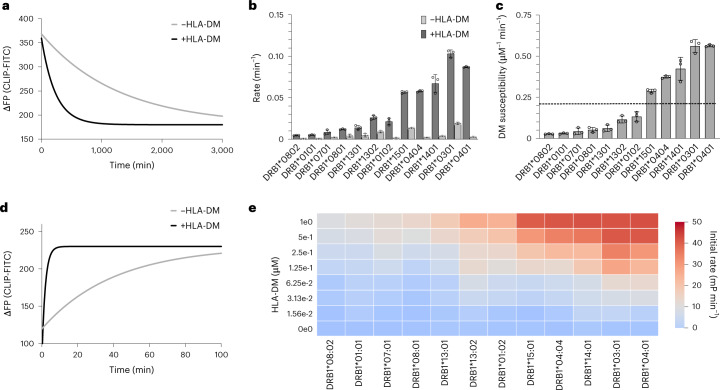
Peptide exchange kinetics of the CLIP peptide for the investigated DRB1 natural variants. **a**, Representative plot of the CLIP-FITC peptide dissociation shown for DRB1*01:01, as detected by a change in fluorescence polarisation (ΔFP). **b**, Dissociation rates (*k*_off_) of the CLIP-FITC peptide measured for 12 DRB1 natural variants in competition experiments in the presence and absence of DM. **c**, DM susceptibility calculated from dissociation experiments as described in Methods. The dashed line represents the average value of DM susceptibility (0.24 µM^−1^ min^−1^), which separates the allotypes into two groups of high or low DM susceptibility. Data in **b** and **c** are presented as mean ± s.d. (*n* = 3 independent experiments). **d**, Representative plot of the CLIP-FITC peptide association shown for DRB1*01:01. **e**, Heat map of the association initial velocities (*k*_on_) of the CLIP-FITC peptide measured for 12 DRB1 natural variants in the presence of different DM concentrations. The data result from at least two independent experiments with at least three replicates in one experiment.

Intrinsic peptide off-rate can also be considered a measure of pMHCII stability^10^. Here, to prevent the additional contribution of a different incoming peptide, we measured the intrinsic CLIP off-rate for all 12 DRB1 allotypes by fluorescence polarization detecting exchange of preloaded fluorescein isothiocyanate (FITC)-labeled CLIP against excess of unlabeled CLIP (Fig. 2 and Supplementary Table 1).

Again, there is no linear correlation between intrinsic CLIP off-rate and thermal stability or predicted CLIP affinity (Supplementary Table 1 and Extended Data Fig. 2e–g). This implies that the sum of several polymorphisms influences thermal stability, predicted CLIP affinity and intrinsic CLIP off-rate in a complex manner, for example with independent contributions to CLIP affinity and structural or conformational stability.

However, in line with the above-mentioned previously published observations for single point mutations, a trend can be observed when pairs of allotypes differ in only a few residues (for example, β86 in DRB1*13:01/*13:02; Fig. 1a): a smaller P1 pocket (β85V/86V in DRB1*13:01) coincides with lower thermal stability, predicted CLIP affinity and intrinsic off-rates than in the cognate allele (DRB1*13:02) with the larger P1 pocket (β85V/86G) (Extended Data Fig. 2). A similar trend is seen when two allotypes (DRB1*04:01/*04:04) differ in only one additional site (residue β71 in the P4 pocket region; Fig. 1a). However, the polymorphism β85A/86V in DRB1*01:02 is associated with higher thermal stability, predicted CLIP affinity and intrinsic off-rates than in DRB1*01:01, where the β85V/86G polymorphism leads to a larger P1 pocket (Extended Data Fig. 2).

Thus, there appears to be a counterintuitive trend between thermal stability or predicted CLIP affinity and intrinsic CLIP off-rate for polymorphisms in the P1 pocket, as polymorphisms leading to faster off-rates still come along with higher thermal stability.

Altogether, these observations imply a complex interplay of different polymorphisms on intrinsic pMHCII stability.

### Polymorphisms in DRB1 affect DM-catalyzed peptide exchange

The exchange of the placeholder peptide CLIP can occur spontaneously but is enhanced by the catalyst DM. Here, we analyzed the CLIP off-rates as described above, but in the presence of DM, for all 12 DRB1 allotypes (Fig. 2a,b and Supplementary Table 1). Based on the intrinsic and catalyzed off-rates, the DM susceptibility^8^ can be calculated (see Methods) as a measure of specific peptide exchange rate enhancement.

**Extended Data Fig. 3:**
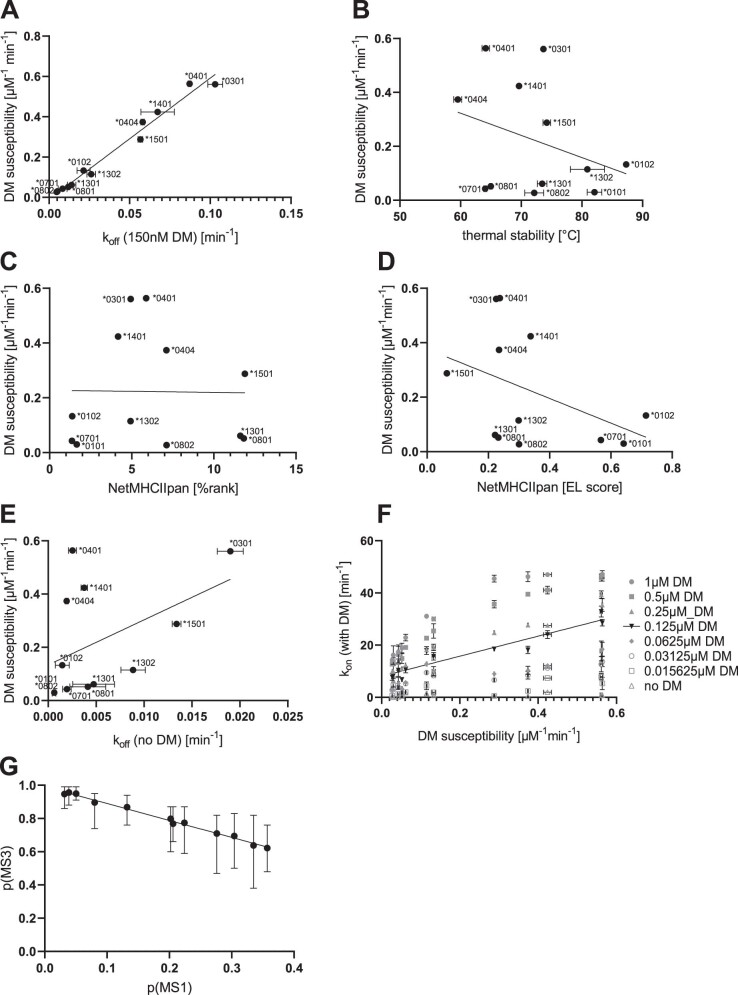
Correlation between parameter of intrinsic stability and catalyzed peptide exchange. Correlation plots are shown for DM susceptibility versus catalyzed (150 nM DM) off-rate (R^2^ = 0.98 **a**), thermal stability (R^2^ = 0.11 **b**), NetMHCIIpan %rank (R^2^ = 0.00 **c**), NetMHCIIpan EL score (R^2^ = 0.18 **d**), intrinsic off-rate (R^2^ = 0.21 **e**). Plotted in **f** are initial apparent on-rates at different DM concentrations against the DM susceptibility for all the analyzed allotypes. As shown in **g**, the population of ground state MS3 shows a negative linear correlation with the population of the DM-susceptible state MS1 (R^2^ = 0.99) in the set of all twelve investigated allotypes. A simple linear regression line is fitted for the data set in the presence of 0.125 µM DM (R^2^ = 0.90). Data for DM susceptibility, thermal stability, on- and off-rates are presented as mean values ± SD (n = 3 independent experiments), error bars for MS state population represent the 1σ confidence interval derived from bootstrapping trajectory MSMs.

Our data show that DM clearly enhances peptide exchange for all of the investigated allotypes, but to a different extent (Fig. 2b,c and Supplementary Table 1). Although DM-catalyzed off-rate correlates well (*R*^2^ = 0.98; Extended Data Fig. 3a) with DM susceptibility, there is no simple linear correlation between parameters of intrinsic pMHCII stability (thermal stability, predicted CLIP affinity and intrinsic off-rate) and DM-catalyzed off-rate or DM susceptibility (Extended Data Fig. 3b–e).

This is in contrast to published observations of a negative linear correlation between the intrinsic stability of the pMHCII complex with catalytic turnover by DM^10^. However, these were measurements with DRB1*01:01 and different peptides, rather than with different allotypes and the same peptide, as in our case. Again, using a pairwise comparison of different allotypes with low numbers of polymorphisms, allotypes with higher intrinsic off-rates indeed show larger DM susceptibility (in Extended Data Fig. 3e, compare DRB1*01:01/*01:02, DRB1*04:01/*04:04, DRB1*13:01/*13:02 and DRB1*08:01/*08:02).

To get further insight into the determinants of DM-catalyzed peptide exchange, we also analyzed apparent on-rates of the CLIP peptide by monitoring binding of fluorescently labeled CLIP to MHC-II preloaded with unlabeled CLIP (see Methods) for all 12 allotypes and, in this case, for different DM concentrations (Fig. 2d,e and Supplementary Table 5). Measured apparent on-rates vary clearly among different allotypes, and there is a strong dependence on DM concentration. Also, although DM susceptibility does not depend on the noncatalyzed apparent on-rates, a linear correlation exists at low DM concentrations (*R*^2^ = 0.90 in the presence of 0.125 µM DM; Extended Data Fig. 3f). At higher DM concentrations, apparent on-rates reach saturation for highly DM-susceptible allotypes (Extended Data Fig. 3f and Supplementary Table 5). Thus, DM-catalyzed apparent on-rates and off-rates are good measures of DM susceptibility of different allotypes. Besides that, measures of intrinsic pMHCII stability do not appear to correlate with the DM susceptibility of different allotypes.

In line with an early suggestion that DM interaction correlates with DR1 flexibility rather than with pMHCII stability^11^, a possible explanation for a more complex scenario would be that polymorphisms influence the conformational equilibrium of the pMHCII complex. We earlier described that DRB1*01:01 in complex with a high-affinity variant of CLIP (CLIP-M107W) adopts a stable ground-state conformation (MS3) as well as a low-populated DM-susceptible conformation (MS1), and we found that the equilibrium can be shifted toward the DM-susceptible state by mutations in the binding groove^5^.

### Population of conformational states varies in DRB1 allotypes

**Fig. 3:**
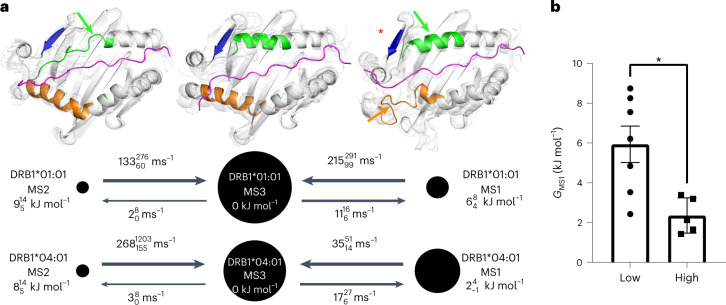
Dynamics of DRB1 natural variants modulate DM susceptibility. **a**, Exemplary MSM kinetic map of DRB1*01:01 and DRB1*04:01. Black discs represent metastable states (ground-state MS3 and two minor states MS1 (DM-susceptible) and MS2) with areas proportional to their relative stationary weights (maximum likelihood values). Black arrows indicate transitions between MSs, where the wider arrows indicate higher corresponding transition rates. Relative free energies and transition rates for the MSs are each shown with a superscript and subscript 1σ confidence interval (from bootstrapping trajectory MSMs). Each MS is illustrated by ribbon representations of MHC-II (white) and CLIP (magenta) of eight simulated conformations (one opaque, seven transparent, viewed from the top into the binding groove). The α-helices of α55–66 and β74-93 are highlighted in green and orange, respectively, with corresponding arrows indicating conformational changes with respect to the ground-state MS3. For localization of the DM interface (indicated by the red star), see Fig. 4. The kinetic maps for all of the other simulated allotypes are shown in Extended Data Fig. 4. **b**, Shown are individual data points of relative free energies (*G*) of MS1 (according to Fig. 3a and Extended Data Fig. 4) for allotypes in groups of low and high DM susceptibility (according to Fig. 2c). Bars represent the mean ± s.e.m. (group of low DM susceptibility (seven allotypes), 5.94 ± 0.92 kJ mol^−1^; group of high DM susceptibility (five allotypes), 2.36 ± 0.40 kJ mol^−^^1^). Data are plotted and analyzed (parametric two-sided *t*-test *P* value = 0.011, *t* = 3.1, d.f. = 10) with GraphPad Prism v9.3.1.

**Extended Data Fig. 4:**
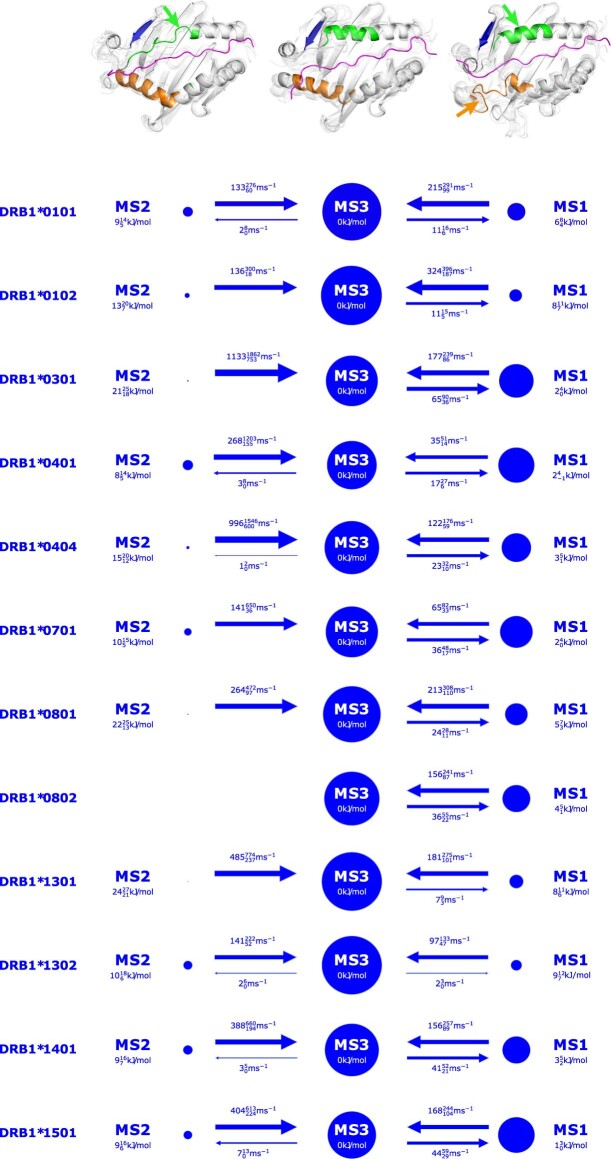
Markov State Model (MSM) kinetic maps of all simulated DRB1 allotypes (twelve allotypes). Black discs represent metastable states (MSs) with areas proportional to their relative stationary weights (maximum likelihood values). Black arrows indicate transitions between MSs, where the width of each arrow increases with the corresponding transition rate. Relative free energies and transition rates for the MSs are each shown with a with a superscript and subscript 1σ confidence interval (from bootstrapping trajectory MSMs). Each MS is illustrated by ribbon representations of MHCII (white) and CLIP (magenta) of eight simulated conformations (one opaque, seven transparent, viewed from the top into the binding groove). The α-helices of α55–66 and β74-93 are highlighted in green and orange, respectively, with corresponding arrows indicating conformational changes with respect to the ground state MS3.

To investigate the conformational equilibrium sampled by the different allotypes, we performed extensive all-atom explicit-solvent molecular dynamics (MD) simulations (Supplementary Table 6). In total, we collected nine millisecond simulation trajectories for all allotypes. This is 15-fold more sampling than in our previous analysis^5^. The simulation data were combined and analyzed by a Markov state model (MSM)^25,26^ using PyEMMA^27^ (see Methods). We found that the DRB1*01:01/CLIP complex samples the same metastable states (ground-state MS3, DM-susceptible state MS1 and excited state MS2; Fig. 3a) as observed for the same allele in complex with the higher affinity CLIP-W peptide^5^. However, with the natural placeholder peptide CLIP, the DM-susceptible state MS1 is clearly more populated (8.0%; Supplementary Table 1) than with the higher affinity peptide (0.06%)^5^. All of the other simulated allotypes were found to sample the same three states, again with differential populations as illustrated for DRB1*01:01 (low DM susceptibility) and DRB1*04:01 (high DM susceptibility) in Fig. 3a and for the other allotypes in Extended Data Fig. 4. For the entire set of allotypes, there is a negative linear correlation (*R*^2^ = 0.99) between the population of the ground-state MS3 and the population of the DM-susceptible state MS1 (Extended Data Fig. 3g and Supplementary Table 1).

**Extended Data Fig. 5:**
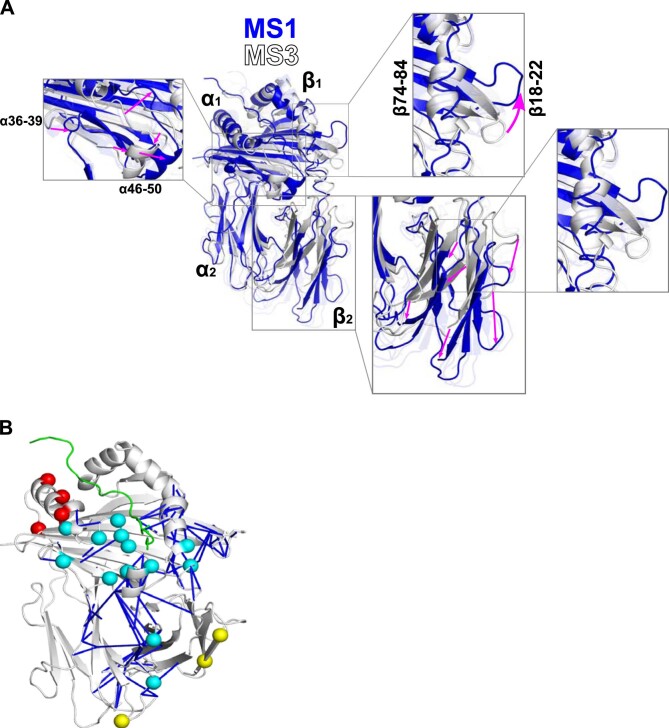
Meta-stable state MS1 exhibits conformational features consistent with DM susceptibility. **a** Significant (2-σ) conformational differences between MS1 (blue ribbons) and MS3 (white ribbons) both sampled uniformly from all simulated allotypes are highlighted by magenta arrows in a superposition of representative MS conformations. **b**. Significant polar interaction frequency differences as derived by PySFD^28^ between MS1 and MS3 are mapped as blue bars onto a representative conformation of MS3, shown in white ribbons (the CLIP peptide is shown in green). These bars form a network of interaction changes that span the entire DM interaction interface on MHCII. Residues, for which it is known from the literature^6,29^ that mutations affect DM-susceptibility, locate to the same region (α40, 43, 49, 51, 53, 54, 55, 57, 61, 96, 100 and β84, 87, 152, 184, 187 shown as cyan spheres) except for three residues (α100 and β184, 187 shown as yellow spheres). Residues, where mutations are known not to affect DM-susceptibility are shown as red spheres (α62, 69, 76, 81) and are outside the network.

As observed before^5^, MS1 shares features of the DM-bound conformation, and the largest differences between MS1 and MS3 conformations locate to regions also differing in a DM-bound crystal structure^6^ (Extended Data Fig. 5a). A PySFD^28^ residual polar interaction analysis between MS1 and MS3 (Extended Data Fig. 5b, blue bars) reveals an allosteric network of significant polar interaction changes. This network comprises the DR/DM interface^6^, and most mutations known^6,29^ to affect DM susceptibility (Extended Data Fig. 5b, cyan spheres) locate to this network. Additionally, the network extends to the DR β-chain and includes polymorphic residues (β25, β26, β73, β74, β77, β78, β85 and β86) implying that polymorphisms located not directly at the DR/DM interface can still trigger changes.

**Fig. 4:**
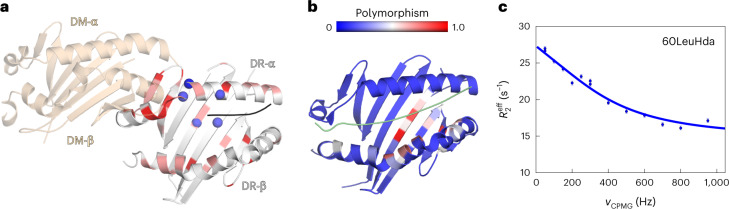
Binding of DM to DRB1 natural variants affects residues in P1-proximal and P1-remote regions. **a**, Peak intensity ratios (*I*/*I*_0_) are mapped on the structure of DRB1*01:01 bound to DM (PDB code 4FQX (ref. ^6^); only the α1 and β1 domains are displayed). Residues of significantly reduced intensity ratios are colored pink if (mean − s.d.) > *I*/*I*_0_ > 0.5, and red if affected even stronger (*I*/*I*_0_ < 0.5) (see also Extended Data Fig. 6). Residues (α10, α59, α60, α65 and β11) showing μs–ms dynamics as derived from ^1^H-^13^C-methyl CPMG relaxation dispersion NMR experiments of DRB1*01:01/CLIP are highlighted as blue spheres (see also Supplementary Table 7). α60, for which the relaxation dispersion curve is displayed in **c**, is highlighted in dark blue. **b**, Global polymorphism analysis of MHC-II proteins. Sequence conservation scores were calculated from DRB1 sequences available in the IPD-MHC Database. The scores were plotted as a blue-to-red spectrum on the structures of DRA/DRB (PDB code 4X5W (ref. ^5^)). **c**, A representative relaxation dispersion curve of methyl groups undergoing conformational exchange $\left( {\Delta \left( {R_2^{\mathrm{eff}},R_2^0} \right) > 2\,{{{\mathrm{s}}}}^{ - 1}} \right)$ in ^1^H-^13^C-methyl CPMG experiments recorded at 27 °C at a ^1^H field of 700 MHz. The effective transverse relaxation rate ($R_2^{\mathrm{{eff}}}$) is shown as a function of the CPMG pulse frequency (*ν*_CPMG_) for one Hd methyl group of the residue α60Leu highlighted in dark blue in **a**.

**Extended Data Fig. 6:**
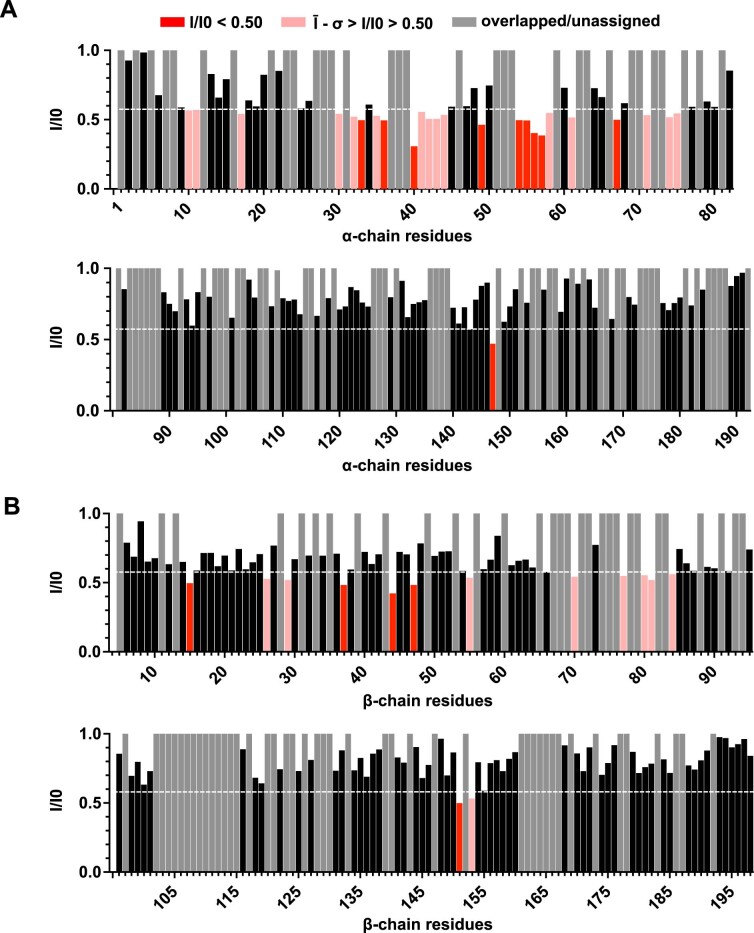
Binding of DM to pMHCII impacts residues in P1-proximate and -remote regions. **a**. and **b**. show peak intensity ratios (I/I0) of backbone amide signals of DRB1*01:01/Cw in the presence and absence of DM. Peaks with an intensity ratio reduced beyond the mean (Ī = 0.72) minus standard deviation (σ = 0.14) (Ī-σ = 0.57, dashed line) are considered significantly affected. Residues of significantly reduced intensity ratios are colored pink if ((Ī-σ)>I/I0 > 0.5) and red if even stronger affected (I/I0 < 0.5). Overlapping and unassigned peaks are shown in gray.

**Extended Data Fig. 7:**
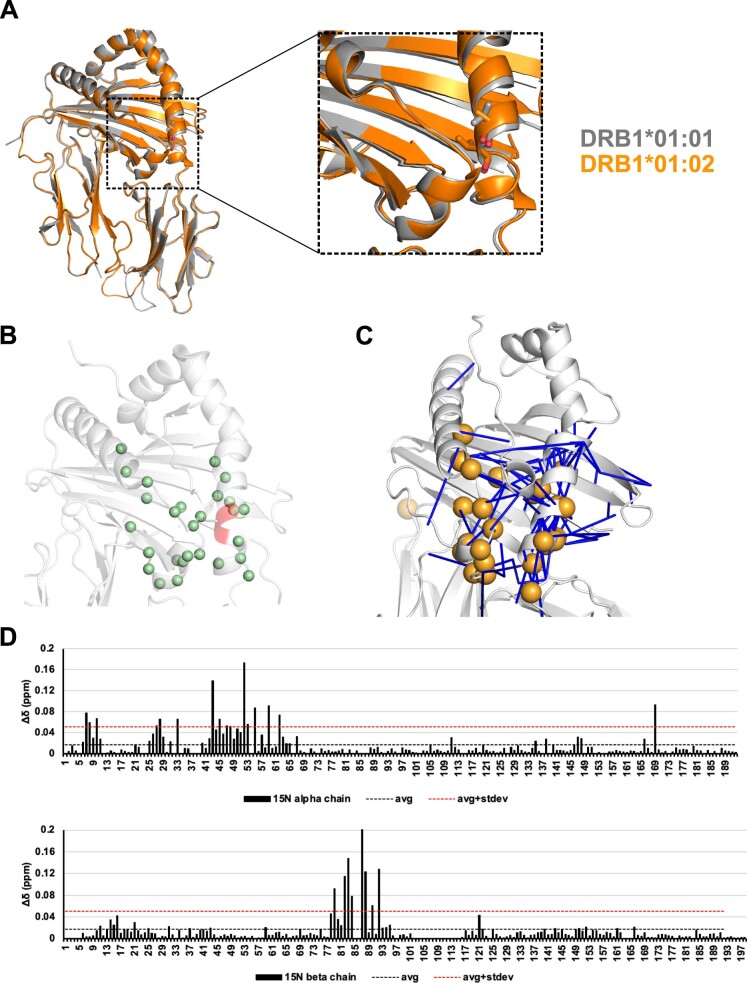
Impact of the natural mutations (V85A, G86V) in the P1 pocket on the structural and dynamic properties of pMHCII proteins. **a**. Left: Structural alignment of DRB1*01:01 (grey, PDB: 3PDO) and DRB1*01:02 (orange). Right: magnification of the N-terminal-binding groove highlighting the natural mutations (V85A, G86V) as sticks. **b**. Cartoon representation of the MHC peptide-binding groove (PDB: 3DPO) with the residues that showed significant chemical shift difference (Δδ(^1^H,^15^N)) in ^1^H-^15^N-TROSY-HSQC spectra of DRB1*01:01 and DRB1*01:02 shown as spheres. **c**. Polar interactions that are significantly more (less) frequent in DRB1*01:01 and DRB1*01:02, as identified with PySFD^28^ and indicated by blue bars mapped on DRB1*01:01 (PDB: 3PDO). **d**. Bar charts of combined chemical shift difference (Δδ(^1^H,^15^N)) between ^1^H-^15^N-TROSY-HSQC spectra of DRB1*01:01 and DRB1*01:02, both co-refolded with CLIP_102–120_. The average plus standard deviation (0.068) is used as significance cutoff in order to define residues highlighted in **B**.

To relate the MS1 population to DM susceptibility, we grouped allotypes for high and low DM susceptibility as indicated in Fig. 2c, then calculated the mean free energy of all MS1 states in each group. Indeed, the group of highly DM-susceptible allotypes showed a significantly lower free energy of the DM-susceptible state MS1 (2.36 ± 0.40 kJ mol^−1^) than the group of low DM susceptibility (5.94 ± 0.92 kJ mol^−^^1^) (Fig. 3b). Thus, the conformational equilibrium of different allotypes partially explains their differences in DM susceptibility. It still indicates that the sum of all polymorphisms contributes in many different ways to the measured biochemical and conformational parameters. As previously suggested^8^, this likely occurs via allosteric communication between the binding groove and the DM binding site. An indication for an extensive dynamic network linking the DM binding site to more distal regions of the binding groove is seen if the DM interaction with DRB1*01:01 is monitored by NMR spectroscopy (Fig. 4a and Extended Data Fig. 6). Upon addition of unlabeled DM to u-^2^H-^15^N-labeled DRB1*01:01 in complex with unlabeled CLIP-W peptide, the observed chemical shift differences were minor (<0.033 ppm), but the loss of signal intensities in the ^1^H-^15^N-TROSY-HSQC spectrum indicates regions of interaction or conformational exchange. The strongest loss in peak intensity is observed for α1 domain residues in the DM binding site, but several residues extending to the opposite site of the binding groove or toward the P4 pocket region, both clearly outside the DM interface, were also significantly affected (Fig. 4a and Extended Data Fig. 6).The fact that the DM interaction induces changes in regions that harbor highly polymorphic residues (Fig. 4b)^1^ indicates that vice versa polymorphisms in the β-chain distal to the DM interface can well affect DM susceptibility by, for example, altering the population of conformational states. Such a scenario is supported by the observation that DRB1*01:01 methyl groups in that region report on dynamics on the µs–ms timescale in ^1^H-^13^C-methyl Carr–Purcell–Meiboom–Gill (CPMG) relaxation dispersion NMR measurements (Fig. 4a, c and Supplementary Table 7), indicating that the center of the binding groove displays the conformational plasticity to adopt different states. Additional evidence for communication along the binding groove is obtained from comparison of the allotypes DRB1*01:01 and DRB1*01:02 that differ only in two residues in the P1 pocket (85V/86G and 85A/86V) and have very similar ground-state crystal structures (Extended Data Fig. 7a and Supplementary Table 4). Comparing NMR spectra shows chemical shift differences, and analyzing the MD simulation data reveals a network of changes in polar interactions, both extending to the P4 pocket region (Extended Data Fig. 7b–d). These findings indicate that allosteric communication pathways extend at least from the P1 to the P4 pocket region.

To further elaborate on cooperativity, we aimed to identify individual residues that are part of an allosteric network capable of translating conformational cues across the binding groove.

### Allosteric coupling across the peptide binding groove exists

**Fig. 5:**
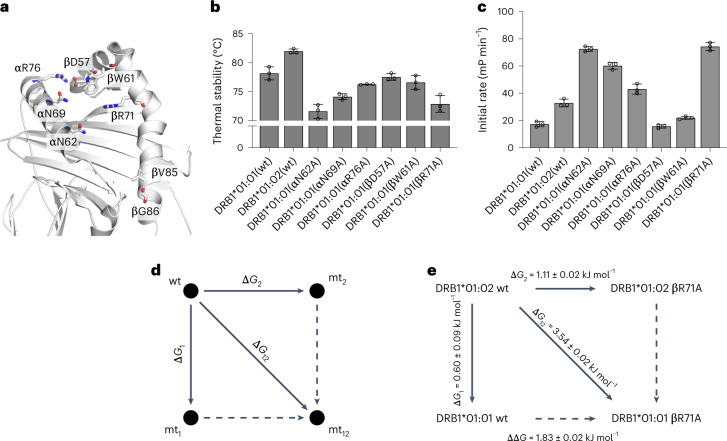
Allosteric coupling through the peptide binding groove modulates stability and peptide exchange in pMHCII. **a**, Representation of DRB1*01:01 (PDB code 3PDO; CLIP peptide is removed for clarity) with the residues mutated into alanine shown as sticks. **b**, Thermal stabilities as detected by thermoshift measurements of the DRB1*01:01/CLIP mutants. **c**, Initial velocities for DM-catalyzed exchange of the fluorescently labeled CLIP-FITC peptide against the CLIP peptide on the variants of DRB1*01:01 at 1 µM DM concentration. Data in **b** and **c** are presented as mean ± s.d. (*n* = 3 independent experiments). **d**, Schematic representation of the double mutant cycle (DMC) for the assessment of the coupling energy between two sites in the MHC peptide binding groove. Given two mutants (mt_1_ and mt_2_), the coupling free energy between them is defined as the extent to which the effect in the double mutant mt_12_ (Δ*G*_12_) is different from the sum of the effects in each of the mutants individually (Δ*G*_1_ + Δ*G*_2_). **e**, DMC analysis is performed on MHC-II variants with DRB1*01:02 as wt; DRB1*01:01 and DRB1*01:02 βR71A as mt_1_ and mt_2_, respectively; and DRB1*01:01 βR71A as mt_12_. All values used in DMCs are listed in Supplementary Tables 8 and 9.

Allosteric networks can be unraveled by the measurement of changes in free energy comparing single and double mutants distal to each other (double mutant cycle)^30,31^. Here, we chose residues across the binding groove that are either highly conserved and involved in the H-bond network with the bound peptide (such as αN62 in the P6 pocket region and αN69/βW61 in the P9 pocket region), or are highly polymorphic (such as β85/β86 in the P1 pocket region and β71 in the P4 pocket region, as well as αR76/βD57 that forms an H-bond at the end of the P9 pocket) (Fig. 5a).

**Extended Data Fig. 8:**
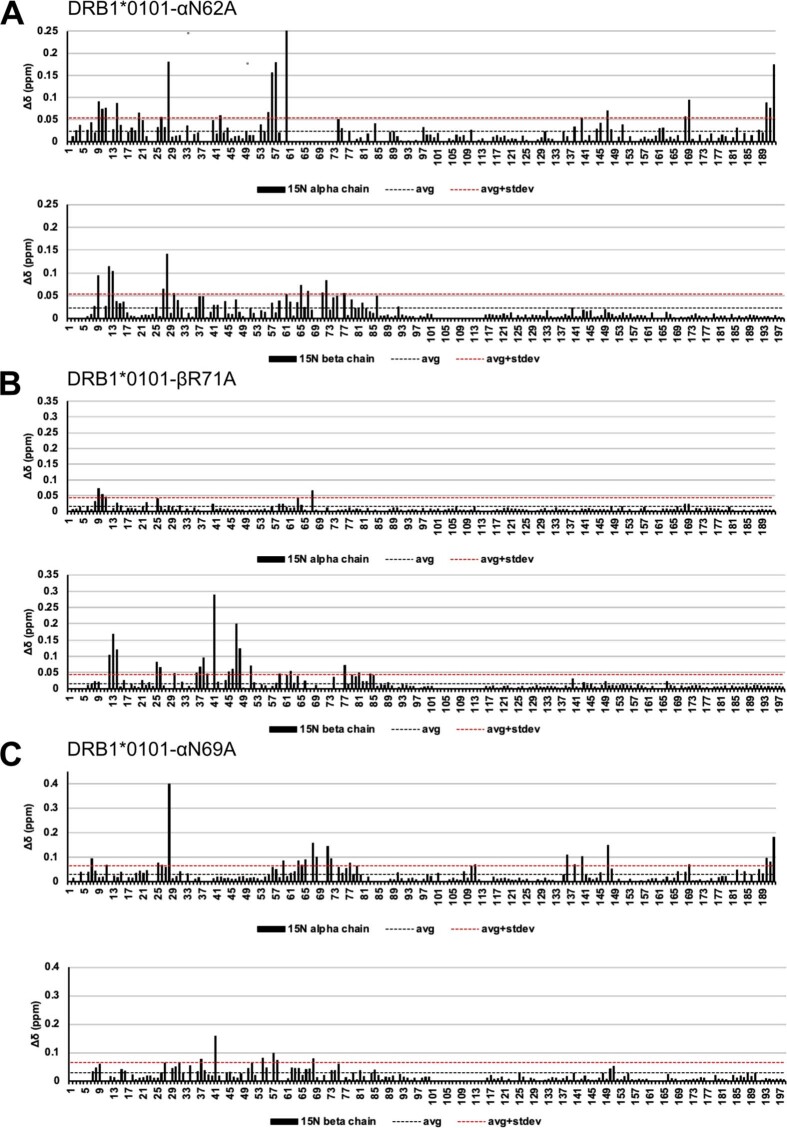
Chemical shift perturbations (Δδ(^1^H,^15^N)) of indicated mutants compared to DRB1*01:01. Bar charts of combined chemical shift differences (Δδ(^1^H,^15^N)) between ^1^H-^15^N-TROSY-HSQC spectra measured at 37 °C of co-refolded DRB1*01:01 and DRB1*01:01-αN62A (**a**), DRB1*01:01-βR71 A (**b**) and DRB1*01:01-αN69A (**c**) for the ɑ (top) and β-chain (bottom), all co-refolded with CLIP_102–120_. The dotted lines indicate the average of all chemical shift differences (black, 0.023(**A**), 0.016(**B**), 0.029(**C**)) and the average plus the standard deviation (red), 0.054(**A**), 0.044(**B**), 0.0649(**C**)).

Introducing the DRB1*01:02 β85/β86 polymorphism or individual alanine substitutions of the other residues mentioned above in the context of DRB1*01:01 results in folded and functionally active proteins as judged from thermal stabilities (Fig. 5b) and DM-catalyzed apparent CLIP on-rates (Fig. 5c). The structure of the pMHCIIs remains overall the same as judged from ^1^H-^15^N-TROSY-HSQC spectra of DRB1*01:02, DRB1*01:01-αN62A, DRB1*01:01-β71A or DRB1*01:01-αN69A (Extended Data Figs. 7 and 8). Rather local changes in the vicinity of the mutations but also extending to the opposite site and along the binding groove are detectable. The individual mutations clearly affect the thermal stability of the pMHCII (Fig. 5b). When the mutation breaks two H-bonds to the peptide (αN62A, αN69A and βR71A), the reduction in thermal stability as compared with wild-type DRB1*01:01 is more pronounced than when only one H-bond is broken either between the peptide and MHC-II (βW61A) or between the two MHC-II chains (αR76A and βD57A). This is again in line with the observation that for single residue changes, the effect on biochemical parameters can be explained with a simple model, such as fewer H-bonds lead to lower CLIP affinity and thus to lower thermal stability. Interestingly, apparent CLIP on-rates are fastest for mutants αN62A and βR71A (Fig. 5c). These mutations break H-bonds in the P4 pocket region, thus highlighting its relevance.

**Extended Data Fig. 9:**
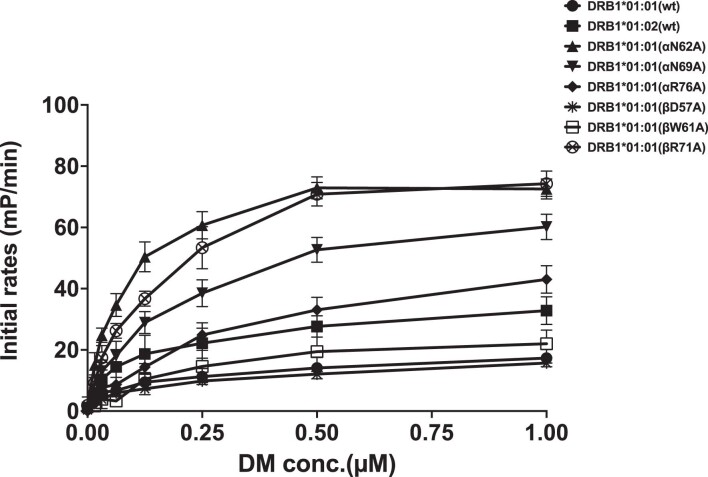
Initial velocities for DM-catalyzed exchange for the alanine mutants of DRB1*01:01. Initial velocities for DM-catalyzed exchange of the fluorescently-labeled CLIP-FITC peptide against the CLIP peptide for the alanine mutants of DRB*01:01 at different DM concentrations. Data are presented as mean values ± SD (n = 3 independent experiments).

We then used the single mutants targeting the P4 pocket region for a double mutant cycle analysis with the polymorphisms in the P1 pocket. Mutations βR71A and αN62A were introduced into either DRB1*01:01 or DRB1*01:02. Dissociation constant (*K*_d_) values were obtained in equilibrium experiments by measuring the fraction of bound fluorescently labeled CLIP peptide in the presence of titrated concentrations of MHC-II (Supplementary Table 8 and Extended Data Fig. 9). From that, interaction free energy (Δ*G*) values were calculated between each pair of pMHCII molecules (Supplementary Table 9). The principle of the double mutant cycle analysis is illustrated in Fig. 5d and shown for the βR71A mutation in the context of DRB1*01:01 and DRB1*01:02 in Fig. 5e.

As the ΔΔ*G* values for βR71A and αN62A in combination with the β85/β86 polymorphism are clearly different from zero (1.83 ± 0.02 and 1.30 ± 0.14, respectively; Fig. 5e and Supplementary Table 9), it can be derived that the P1 and P4 pocket regions are energetically coupled.

Thus, allosteric communication between the P1 and P4 pocket regions probably underlies the complex effect of polymorphisms on the biochemical parameters and the conformational landscape that we observe.

### The DM-susceptible state contributes to disease association

**Fig. 6:**
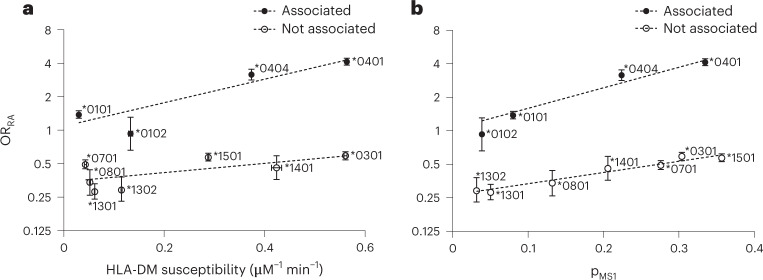
Correlation of odds ratio for rheumatoid arthritis with DM susceptibility and MS1 state population. **a**, **b**, Plotted is the odds ratio (OR) with 95% confidence intervals for rheumatoid arthritis (RA) as derived from the genetic association study in ref. ^34^ against the experimentally determined DM susceptibility (presented as mean ± s.d. (*n* = 3 independent experiments)) (**a**) and population of the MS1 (DM-susceptible) state (**b**) for all 12 DRB1 allotypes. Data are fit in groups of rheumatoid arthritis-associated and not associated allotypes (**a**, *R*^2^_associated_ = 0.91 and *R*^2^_not associated_ = 0.49; **b**, *R*^2^_associated_ = 0.95 and *R*^2^_not associated_ = 0.94).

Above, we unraveled a composite effect of all polymorphisms on the different determinants of antigen exchange: CLIP affinity, pMHCII stability and DM susceptibility. Given that DM has an effect on the development of autoimmune diseases^32^, it can be hypothesized that the DM susceptibility of an allele—even if kept within a certain range—could also contribute to the risk of disease. To expand on that, we looked at the association of DR1 allotypes and rheumatoid arthritis: a positively charged P4 pocket, which enables the presentation of citrullinated peptides, is known to be associated with higher risk for rheumatoid arthritis, which in turn is often characterized by the presence of autoantibodies against citrullinated proteins. The presence of the so-called shared epitope^33^ in DRB1 (QKRAA, QRRAA or RRRAA in residues β70–β74) that renders the P4 pocket positively charged is used to support the diagnosis of rheumatoid arthritis. It has been found by statistical analysis on the amino acid level that polymorphisms at DRB1 β11 (V, L), β71 (K) and β74 (A) account for most of the association with rheumatoid arthritis^34^. In our data set, the allotypes DRB1*01:01, DRB1*01:02, DRB1*04:01 and DRB1*04:04 contain the shared epitope and above-mentioned risk polymorphisms (Fig. 1a; DRB1*04:01, _70_QKRAA_74_/β11V; DRB1*04:04, _70_QRRAA_74_/β11V; DRB1*01:01 and DRB1*01:02, _70_QRRAA_74_/β11L). Consequently, the odds ratios for these allotypes are higher than those for allotypes not carrying the shared epitope, reflecting the genetic association of these alleles with disease^34^ (Supplementary Table 1). However, the odds ratio of allotypes with shared epitope and risk polymorphisms varies between 0.93 (DRB1*01:02) and 4.14 (DRB1*04:01), indicating that the presence of a suitable P4 pocket alone does not explain risk for disease. We hypothesized that—in line with our results—DM susceptibility might contribute to disease association. Therefore, we plotted the odds ratio for rheumatoid arthritis as previously determined^34^ against DM susceptibility for the 12 investigated allotypes (Fig. 6a). Apparently, the four allotypes with the shared epitope show a strong positive dependence of the odds ratio on DM susceptibility, whereas the other allotypes remain protective for rheumatoid arthritis. Moreover, as DM susceptibility is linked to the population of the MS1 state (Fig. 3), we also plotted the odds ratios against the MS1 occupancy (Fig. 6b). Again, a correlation is seen between higher MS1 population and increased odds for disease, this time both in the associated set and the protective set of allotypes (Fig. 6b). This indicates that the modulation of DM-catalyzed peptide exchange by natural polymorphisms in DRB1 allotypes contributes to disease associations.

## Discussion

Here, we investigated the effect of natural polymorphism on parameters of peptide exchange in a set of 12 DRB1 allotypes in complex with the natural placeholder peptide CLIP. This analysis compares a large set of highly abundant (Extended Data Fig. 1a) DRB1 allotypes. We observed that intrinsic stability, DM-catalyzed peptide exchange and population of conformational states differ among pMHCII allotypes. An allosteric network exists that energetically links the P1 and P4 pocket regions and allows the translation of the effect of polymorphisms across the binding groove to induce conformational changes also at the DM binding site.

Distal communication through the MHC-II peptide binding groove has been shown previously by mutating individual peptide anchor residues or conversely MHC-II residues that shape the pockets or form H-bonds to the peptide^5,8,10^. For example, suboptimal pocket occupation in the context of DRB1*01:01 was shown to differentially affect intrinsic pMHCII stability in correlation with catalytic turnover by DM^10^. In the context of DRB1*03:01, it has been shown that mutations in the P4 or P6 pocket region increase the kinetic stability of CLIP–MHC-II complexes, thereby reducing DM-mediated CLIP exchange^35^.

Overall, the MHC-II allotypes studied here differ in many positions rather than in individual positions, so a simple correlation between parameters of intrinsic stability and catalyzed peptide exchange could not be observed among the entire data set, illustrating a more composite effect of these polymorphisms on the parameters of peptide exchange.

**Extended Data Fig. 10:**
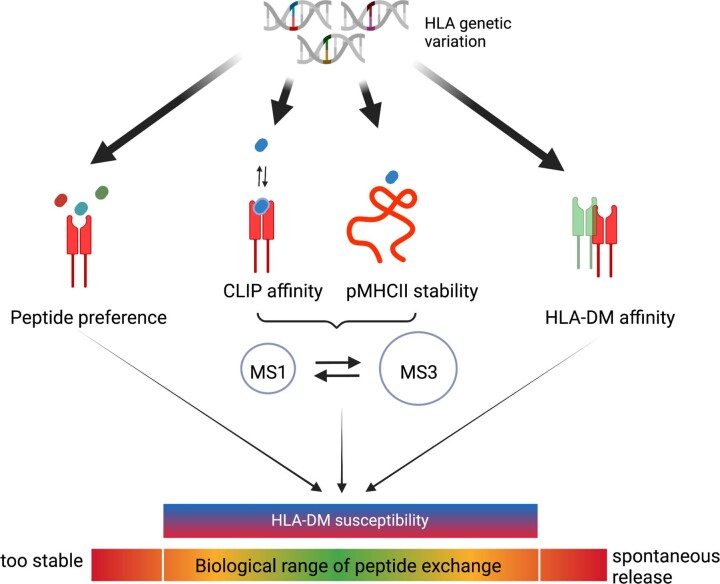
Scheme illustrating the impact of MHC-II natural polymorphism on antigen presentation. Scheme illustrating that polymorphisms in MHC-II influence peptide preference, CLIP affinity, pMHCII stability and DM affinity, which are not independent from each other. CLIP affinity and pMHCII stability influence the conformational equilibrium of CLIP–MHC-II complexes resulting in different populations of MS3 (ground) and MS1 (DM-susceptible) states for all investigated allotypes. All these parameters influence DM susceptibility and ensure that all tested allotypes stay within a neither too fast nor too slow range of peptide exchange. Created with BioRender.com.

These composite effects are likely to influence pMHCII’s conformational landscape^5,9^, which was previously hypothesized to define DM susceptibility^5,8,11–14^. CPMG experiments of the DRB1*0101 allotype presented here confirm the presence of conformational dynamics in the DM binding site. Comparable CPMG data of different allotypes would be a valuable addition, but are currently hindered by the limited amounts of functional protein obtained from bacterial expression. In line with that, most of the earlier publications on DM susceptibility^5,8,11–14^ inspect only the DRB1*01:01 allotype, preventing conclusions at the alleleic level. The results from MD simulation capitalize on the presence of the stable ground-state MS3 and the DM-susceptible state MS1 previously observed for a DRB1*01:01/CLIP-W complex^5^. Interestingly, all allotypes retain the capability to sample the same conformational states MS3 and MS1, but CLIP affinity and pMHCII stability alter the occupancy of states of the individual allotypes. Despite a pronounced effect on pMHCII thermal stability and peptide anchor residue preference, we suggest that combinations of polymorphisms have evolved to keep the MS3/MS1 equilibrium in a range that allows for peptide exchange to happen in a biologically suitable window, avoiding spontaneous loss of peptides as well as complexes that are too stable to exchange peptides under physiological conditions (Extended Data Fig. 10).

We further hypothesized that variability in the DM susceptibility of an allotype might have a functional effect with regard to self-peptide presentation. MHC-II allotypes with the ability to present certain self-peptides are genetically associated with autoimmune disease^36^. For example, rheumatoid arthritis is associated with certain DR1 allotypes (in our data set, DRB1*01:01, DRB1*01:02, DRB1*04:01 and DRB1*04:04) characterized by polymorphisms in the P4 pocket^33,34^. Here, we linked the previously determined^34^ association of DRB1 allotypes with rheumatoid arthritis to the allotypes’ DM susceptibilities and MS1 state populations obtained in this study. We find that higher DM susceptibility appears to additionally contribute to higher propensity for disease (Fig. 6a). This is in line with the hypothesis that risk for disease is influenced by not only the likelihood to present certain self-peptides, but also the DM activity^32,37–39^.

The association of higher DM susceptibility with higher risk for disease observed here might especially apply in situations where presentation of self-peptides depends on DM catalysis, for example in situations of low abundance of a high-affinity self-peptide. In other scenarios, editing by DM may be required to restrain self-reactivity as has been shown for HLA-DQ2, an MHC-II allotype associated with celiac disease. The low DM susceptibility of the HLA-DQ2 allotype leads to the presentation of autoimmunogenic gliadin peptides that would be otherwise removed by DM^40,41^.

Taken together, we show that polymorphisms in DRB1 allotypes act in a composite manner on parameters of peptide exchange. Allosteric networks exist that translate polymorphisms in changes of the conformational landscape of pMHCII, thereby altering the population of a DM-susceptible state. Even the small sample of 12 DRB1 allotypes out of thousands of HLA-DR, HLA-DQ and HLA-DP variants already reflects a range of different peptide-exchange behaviors. Among those allotypes not investigated here, there are probably extreme cases that are resistant to DM catalysis^40^ or easily lose CLIP^42^. However, the observations that even DR allotypes of low thermal stability display only limited intrinsic CLIP off-rates and highly thermostable allotypes are still susceptible to DM-catalyzed exchange supports the hypothesis that polymorphisms need to resolve in a target range of intrinsic stability and DM susceptibility.

## Methods

### Expression and purification of DR and DM

DR natural variants were expressed and purified using the baculovirus–insect cell expression system (Bac-to-Bac baculovirus expression system and the pFastBac Dual vector (Thermo Fisher)) encoding for the CLIP peptide fused to the N terminus of DRB1 chains including a thrombin cleavage site and DRB1 chains including C-terminal leucine zippers to improve stability as previously described^18^. First, the constructs based on pFastBac Dual were used to transform DH10Bac cells (Thermo Fisher Scientific) and to produce bacmids according to the manufacturer’s specifications. Next, the virus was amplified upon transfection and infection of Sf9 cells (Thermo Fisher). For protein expression, Sf9 cells in the exponential growth phase (1–2 × 10^6^ cells ml^−1^) were infected at a multiplicity of infection of 10, then kept at 27 °C for 3–4 days. Proteins were purified from the supernatants by immunoaffinity chromatography using the antibody L243 (HB-55, ATCC) coupled to Sepharose Fast Flow (Cytiva).

The purified proteins were treated with thrombin (20 U mg^−1^ in PBS; Sigma-Aldrich) to get noncovalently linked peptide, and cleavage was verified by the shift of β-chain mobility on SDS–PAGE. The reaction mixtures were gel-filtrated using Superdex S200 (GE Healthcare); multimeric complexes and aggregates were discarded. Fractions containing the proteins of the correct size were pooled and concentrated using Vivaspin 30 kDa MWCO spin filters.

DRA*01:01 and DRB1*01:01 (residues 1–192 and 1–198, respectively^17^) subunit-derived constructs were generated by site-directed mutagenesis using the standard Quick Change protocol to introduce mutations. Both protein chains were individually expressed in *Escherichia coli*. They were purified from inclusion bodies under denaturing conditions by ion exchange and co-refolded in the presence or absence of CLIP peptide by dilution^5,17^. After concentration proteins were affinity-purified as described above. DM (DMA1*01:01 and DMB1*01:01) was cloned into the pFastBac Dual vector^18^. The original C-terminal protein C tag in the β-chain was replaced with a biotin acceptor sequence, and the α-chain Flag-tag was used for purification. DM was produced using the above-mentioned baculovirus–insect cell expression system (pFastBac Dual Sf9). Bacmids were produced, virus was amplified and DM was expressed as described above for DR. DM was purified from the supernatant by immunoaffinity chromatography and M2 Sepharose (Sigma-Aldrich).

### Thermoshift measurements

After linker cleavage and size-exclusion chromatography (as mentioned above, final concentration of 0.4 mg ml^−1^), monomeric pMHCII protein was mixed with 5× Sypro Orange (Life Technologies). Using quantitative PCR (qPCR; Mx3005P, Stratagene), the temperature was increased for 2 °C min^−1^, and the emission was detected at 575 nm in lifetime after exciting the dye at 490 nm. Fluorescence intensity was plotted versus the temperature, and a sigmoidal function was fitted to determine the midpoint temperature of the unfolding reaction (thermal stability, °C). Stability measurements were performed in PBS buffer at pH 5.8. For the comparison of thermal stabilities of 0.4–0.75 mg ml^−1^ (~7–14 µM) DRB1*01:01 or DRB1*04:01 in the absence or presence of 1 mM CLIP peptide, we used the qPCR machine (StepOnePlus, Applied Biosystems). All graphs—including those resulting from the subsequent methods—were plotted using GraphPad Prism v9.3.1, and figures were designed using Inkscape v0.91 and v1.1.

### Fluorescence polarization assay

Association of 100 nM CLIP_102–120_ (KPVSKMRMATPLLMQALPM) labeled with FITC (CLIP-FITC) on 1 μM DR/CLIP_102–120_ complexes was followed by fluorescence polarization on a Victor 3V (PerkinElmer) or Tecan Spark plate reader in the presence or absence of titratable concentrations (2-fold dilutions) of DM at 25 °C in order to derive apparent on-rates (*k*_on_). Peptide dissociation of 100 nM DR/CLIP-FITC complexes was monitored in the presence and absence of 20 μM competitor peptide (CLIP_102–120_) and 150 nM DM at 37 °C. All exchange reactions were set up in triplicates of 40 μl in phosphate/citrate buffer at pH 5.2 as previously described^18^. DM susceptibility is calculated as (*k*_off,DM_ − *k*_off,in_)/[DM], where *k*_off,DM_ is the DM-catalyzed off-rate, *k*_off,in_ is the intrinsic off-rate and [DM] is the concentration of DM used in the experiment.

For equilibrium experiments, concentrations of empty DR as derived from bacterial expression were varied from 4 μM to 7.8125 nM (2-fold dilutions) with a fixed CLIP-FITC concentration of 100 nM. Dissociation constants (*K*_d_) were determined, and the interaction free energy (Δ*G*) values between each pair (*i*, *ii*) of DR/CLIP-FITC complexes in the double mutant cycles were calculated as $\Delta G = RT\ln \frac{{K_{{\mathrm{d}}_{ii}}}}{{K_{{\mathrm{d}}_{i}}}}$.

### CLIP binding prediction by NetMHCIIpan

NetMHCIIpan-4.0^23^ (accessed February 2022) was used to predict binding of the CLIP peptide to DRB1 allotypes. NetMHCIIpan prediction algorithms are trained on data sets of in vitro binding affinities and mass spectrometry of MHC-II-eluted ligands^24^. The 15-mer peptide CLIP_103–116_ (PVSKMRMATPLLMQA) was used. For all analyzed allotypes, the core motif MRMATPLLM was the strongest binding core placing Met107 in the P1 pocket and Met115 in the P9 pocket. Both output measures are reported: the eluted ligand mass spectrometry (EL) score for likelihood of binding and the %rank score normalized to a set of random peptides. The higher the EL score and the lower the %rank value, the higher the affinity of the peptide.

### Nuclear magnetic resonance (NMR)

NMR spectra were acquired on a Bruker Avance III 700 MHz spectrometer equipped with a 5-mm triple resonance cryoprobe. Spectra were processed with TopSpin v3.2 (Bruker) and analyzed with CcpNmr Analysis v2.4.2^43^. The NMR measurements were performed at 310 K (unless otherwise stated) in PBS buffer at pH 5.8 containing 10% D_2_O with a protein concentration of 28–360 μM. To reduce the spectral complexity, only one of the chains was ^15^N-labeled (unless otherwise stated) at a time. Backbone assignments of DRB1*01:01/CLIP_102–120_^21^ were transferred to the mutants resulting in the assignment of approximately 90% α-chain and 80% β-chain ^1^H-^15^N-TROSY-HSQC resonances for all DRB1*01:01 mutants. For ^15^N-β-labeled DRB1*01:02-βR71A/CLIP and ^15^N-α-labeled DRB1*01:02-αN62A/CLIP, transferred assignments were confirmed by ^1^H-^15^N-NOESY-HSQC spectra.

Chemical shift perturbation was calculated according to the following equation.

$$\Delta \delta \left( {{\,}^1{\mathrm{H}}^{15}{\mathrm{N}}} \right) = \sqrt {\left( {\delta \left( {{\,}^1{\mathrm{H}}} \right)} \right)^2 + \left( {0.15\Delta \delta \left( {{\,}^{15}{\mathrm{N}}} \right)} \right)^2}$$

To investigate the interaction of DR1 with DM, we measured ^1^H-^15^N-TROSY-HSQC spectra with 160 scans of 150 μM ^2^H−^15^Nαβ-labeled DRB1*01:01/CLIP_102–120_M107W in the presence and absence of 75 µM of DM. Peak intensity ratios (*I*/*I*_0_) were calculated from spectra in the presence (*I*) and absence (*I*_0_) of DM.

### CPMG relaxation dispersion

CPMG relaxation dispersion experiments of 150–350 μM ^1^H-^13^C-ALV-^15^N-α-U-^2^H DRB1*01:01/CLIP and ^1^H-^13^C-AILV-^15^N-β-U-^2^H DRB1*01:01/CLIP were recorded single-scan interleaved using an insensitive nuclei enhancement by polarization transfer (INEPT) for excitation^44^ and a WATERGATE element for water suppression in order to measure in 10% D_2_O. CPMG pulse frequencies of 0, 950, 50, 300, 150, 800, 100, 250, 400, 600, 200, 50, 500, 300 and 700 Hz with a constant time delay of 40 ms were used. Peak intensities were converted to the *R*^eff^ transverse decay rates with the equation $R_2^{{\mathrm{eff}}} = \frac {1} {T_{{\mathrm{CPMG}}}} \times \ln \frac {I_0} {I_{{\mathrm{CPMG}}}}$. Only assigned and nonoverlapping peaks were analyzed. CPMG profiles of all methyl groups displaying dispersion $\left( {\Delta \left({R_2^{{\mathrm{eff}}},R_2^0} \right) > 2\,{\mathrm{s}}^{ - 1}} \right)$ were fitted to a two-state model using the program NESSY^45^.

### Global polymorphism analysis

The HLA sequences for the DR gene were acquired from the IPD-IMGT/HLA Database^46^. For MHC-II, this included 29 HLA-DRA and 2,638 HLA-DRB1 sequences. The sequences were aligned, and the entropy score value of each position was calculated as $C_{{\mathrm{entropy}}} = \frac{{ - \mathop {\sum}\nolimits_\alpha ^K {\rho _\alpha } \mathop {{\log }}\nolimits_2 \rho _\alpha }}{{\mathop {{\log }}\nolimits_2 \left( {{{{\mathrm{min}}}}(N,K)} \right)}}$, where *N* is the number of residues in each sequence position, *K* is the number of residue types, *ρ*_α_ is *n*_α_/*N*, and *n*_α_ is the number of residues of type α.

### Molecular dynamics (MD) setup and simulations

To prepare DRB1 allotype-specific starting conformations for MD simulation, we first resorted to our combined set of MD simulations of DR1/CW wild type and the βN82A (P1 pocket) mutant (an aggregate of about 600 μs), previously performed as regular MD in ref. ^5^, and prolonged with an adaptive MD protocol developed in ref. ^10^. This data set was then projected with time-independent component analysis (TICA; lag time *τ* = 75 ns) onto the three most important time-independent components (TICs) and clustered in the space spanned by these three TICs with *k-*means with 500 cluster centers. As a result, we obtained sets of discretized (microstate) simulation trajectories, on which we performed Bayesian MSMs (BMSMs) at a lag time of 75 ns on each set of the wild-type and mutant simulations. These BMSMs were then used to select starting conformations uniformly along the slowest simulated dynamic processes. For each BMSM of DR1/CW and DR1-N82A/CW, we first clustered the corresponding discrete microstate trajectories in the projected space of the second to fourth BMSM eigenvectors, using 9 and 15 *k*-mean clusters for the DR1/CW and DR1-N82A/CW BMSMs, respectively (these numbers of clusters were chosen to ensure sufficient cluster center coverage of the eigenvector space; that is, each contour region plotted along each pair of eigenvectors had to be represented by at least one cluster center to allow sampling in that region). From each of the two cluster sets (DR1/CW and DR1-N82A/CW), first a cluster was uniformly sampled to identify a corresponding microstate, then a corresponding simulation frame was uniformly sampled from each microstate. We repeated this procedure 125 times for each of the two cluster sets, resulting in 250 starting conformations.

Each allotype was then modeled by introducing clash-free point mutations via high-throughput molecular dynamics^47^ using PDB2PQR^48,49^ into each of the 250 conformations, with the determined residual protonation.

Protonation states for each modeled allotype were estimated by the sampled 250 conformations above. Each allotype was then modeled twice by introducing point mutations into each of the 250 conformations of each BMSM. Using the program PROPKA v3.1^50^, the average p*K*_a_ values of all titratable side chains were determined for each allotype model, with which protonation states at a MHC-II physiological pH of 5.8 were assigned. For each allotype, all protonation assignments were consistent among the DR1/CW-based and DR1-N82A/CW-based models. Upon validation of all these protonation states, we noticed that PROPKA had assigned several residues (Glu21, Asp25, Asp29 and Asp66) to be completely ‘buried’ and thus to be protonated. As in DR1 crystal structures (PDB codes 3PDO and 4FQX), as well as in our MD simulations, these residues are solvated by at least two water molecules on average (even when protonated in MD), therefore we decided to not protonate these residues here.

The actual MD simulations on each allotype were performed as previously described^5^, that is, with the ACEMD^51^ software using the ff99SB63 Amber force field^52^, an integration time step of 4 fs and a hydrogen mass scaling factor of 4, a 1–4 scaling factor of 5/6 orthorhombic periodic boundary conditions and particle-mesh Ewald electrostatics with 1 Å grid spacing, a 9 Å cutoff, switching at 7.5 Å, scale 1–4 exclusion, full electrostatic frequency of 2 steps, fixed bonded interactions between heavy atoms and hydrogens (‘rigidbonds all’), and Langevin dynamics. For further details, see ref. ^5^.

### MSM analyses

MSM analysis was performed by selecting as input features the pairwise distances of all Cα atoms within 15 Å of the β82 location in a simulated DR1 crystal structure (PDB code 3PDO)^21^; that is, αH5-αA10, αM23-αD27, αE30-αF32, αF48-αE55 (not αR50), βR13-βL27 (not βN19) and βR71-βR93 (not βR72 and βΝ82) were used as input coordinates to a time-lagged independent component analysis, exactly as in ref. ^5^. A table containing all of these contacts is available upon request.

Each MSM analysis was performed by TICA^53,54^ on the input features of the joint simulation set, and *k*-means clustering in the space of the first (2–4) TICA dimensions to define consistent microstates among simulation conditions. To also define consistent microstate-to-metastable state assignments, we built a consensus MSM over the microstate trajectories of all considered simulations, followed by a PCCA+ analysis^55^; the number of metastable states was defined by the maximum that would still give sufficient metastable populations after clear metastable assignment filtering (cutoff, 50%).

These assignments were later used for the estimation of all individual MSMs of each simulated condition, so that each individual MSM is built on microstates and metastable states that are both consistent between simulated conditions. The detailed parameters of the set of 12 DRB1 allotypes are as follows: TICs with a lag time of 2,250 ns, 500 *k*-mean clusters, and an MSM lag time of 250 ns.

### Crystallization and data collection

Insect cell-expressed DRB1*01:02, DRB1*04:01 and DRB1*07:01, all in complex with the CLIP_102–120_ peptide, were concentrated to 10 mg ml^−1^ in buffer containing 20 mM MES at pH 6.4 and 50 mM NaCl. All crystals of the DRB1 complexes were grown at 293 K using the sitting-drop vapor-diffusion method by mixing 0.2 µl of protein solution with 0.2 µl of precipitant solution. The composition of the precipitant solution for the DRB1*01:02/CLIP complex was 25% PEG 3350, 0.2 M MgCl_2_ and 0.1 M HEPES pH 7.5. The precipitant solution for the DRB1*04:01/CLIP complex contained 20% PEG 3350, 0.2 M ammonium dihydrogen citrate, and the precipitant solution for the DRB1*07:01/CLIP complex contained 20% PEG 3350, 0.15 M sodium malonate. Crystals suitable for X-ray diffraction grew for all complexes within 2–10 days. We cryoprotected the crystals for data collection by soaking them for a few seconds in their precipitant solution with the addition of 10% ethylene glycol and subsequently froze them in liquid nitrogen.

Diffraction data to a resolution of 2.1 Å were collected for the DRB1*04:01/CLIP and DRB1*07:01/CLIP complexes and to a resolution of 1.76 Å for the DRB1*01:02/CLIP complex at 100 K at beamline BL14.1 at the synchrotron radiation source BESSY II (Helmholz-Zentrum Berlin) and processed using the XDSAPP suite^56^.

Figures displaying crystal structures were prepared using PyMOL v1.8.0.0 (Schrödinger, LLC). RMSD values for Cα atoms were calculated between structures solved here and the DRB1*01:01/CLIP structure (PDB code 3PDO (ref. ^21^)) using the cmd.align command in PyMOL.

### Structure determination and refinement

The crystal lattice of the DRB1*01:02/CLIP complex belongs to the primitive monoclinic space group P2_1_. The diffraction patterns for DRB1*04:01/CLIP could be indexed in the C-centered orthorhombic lattice C222_1_ and for the DRB1*07:01/CLIP in primitive rhombohedral lattice R3. Using the structure of the DR1 mutant βN82A with deleted peptide (PDB code 4X5X) as a starting model, a solution could be found for the DRB1*01:02/CLIP and DRB1*04:01/CLIP data by molecular replacement with the program Phaser^57^ with two complexes per asymmetric unit. The DRB1*01:02/CLIP and DRB1*04:01/CLIP complex structures were refined with PHENIX^58^ to final crystallographic *R*_work_*/R*_free_ values of 16.7/20.3% and 18.7/22.2%, respectively. The DRB1*07:01/CLIP complex structure was also solved by molecular replacement with Phaser, using the DRB1*04:01/CLIP complex structure without the CLIP peptide as a search model. One complex per asymmetric unit could be found, and the DRB1*07:01/CLIP complex was refined with BUSTER^59^ to a final crystallographic *R*_work_*/R*_free_ value of 21.5/24.3%. Structure validation was performed with MolProbity^60^. The crystallographic data and refinement statistic are listed in Supplementary Table 3.

### Reporting summary

Further information on research design is available in the Nature Portfolio Reporting Summary linked to this article.

## Online content

Any methods, additional references, Nature Portfolio reporting summaries, source data, extended data, supplementary information, acknowledgements, peer review information; details of author contributions and competing interests; and statements of data and code availability are available at 10.1038/s41589-023-01316-3.

### Supplementary information

Supplementary Information Supplementary Tables 1–9, Supplementary References.

Reporting Summary

## Data Availability

Crystal structures are deposited to the PDB for DRB1*01:02 (PDB code 7YX9), DRB1*07:01 (PDB code 7Z0Q) and DRB1*04:01 (PDB code 7YXB). Other data are available from the corresponding author upon reasonable request.
