# Supplementary material for: MHC-II dynamics are maintained in HLA-DR allotypes to ensure catalyzed peptide exchange
Source: Nat Chem Biol. 2023 May 4;19(10):1196–204. doi: 10.1038/s41589-023-01316-3 (PMC10522485; doi:10.1038/s41589-023-01316-3)
Supplement: Supplementary file 1 — Supplementary Tables 1–9, Supplementary References. [file 41589_2023_1316_MOESM1_ESM.pdf]

nature chemical biology

Article https://doi.org/10.1038/s41589-023-01316-3

MHC-II dynamics are maintained in HLA-DR allotypes to ensure catalyzed peptide exchange

In the format provided by the authors and unedited

Table of contents

Supplementary Table 1: Experimental and computational as well as previously published data collected for the analyzed DRB1 allotypes…………………………………………………………………2

Supplementary Table 2: Thermal stability of DR1*01:01 and DR1*04:01 in the absence or presence of a ~100fold molar excess of CLIP peptide. Errors bars represent the standard deviation calculated from three (DR1*01:01) or four (DR1*04:01) independent experiments. Measurements were performed on the qPCR maschine StepOne Plus™ (Applied Biosystems)……………………………….…….3

Supplementary Table 3: Crystallographic data collection and refinement statistics…………………..4

Supplementary Table 4: Root mean square deviation (RMSD) values of Cα atoms calculated for crystal structures of CLIP-bound DRB1*01:02 (PDB 7YX9), DRB1*04:01 (PDB (7YXB), and DRB1*07:01 (PDB 7Z0Q) in comparison to CLIP-bound DRB1*01:01 (PDB 3PDO). RMSD values are shown for the entire pMHC complex, the α1β1-domains forming the peptide binding groove of the MHC protein, and for the core residues (P1-P9) of the CLIP peptide…………………………………...5

Supplementary Table 5: Initial apparent on-rates for all analyzed allotypes at different HLA-DM concentrations……………………………………………………………………………………...6

Supplementary Table 6. Overview of molecular dynamics (MD) simulations performed for the DRB1 allotypes in complex with CLIP peptide……………………………………………......................7

Supplementary Table 7: Results of ^{1}H-^{13}C-methyl-CPMG analysis for DR1*01:01 residues……..….8

Supplementary Table 8: Values of the equilibrium dissociation constants (K_D) used in the double mutant cycle calculations of HLA-DR*01:01 mutants. Errors represent the standard deviation and are calculated from three independent experiments……………………………………………………9

Supplementary Table 9: Interaction free energies of the double mutant cycles (all units in kJ/mol). Errors represent the standard deviation and are calculated from three independent experiments……….9

Supplementary references……………………………………………………………………..9

Supplementary Table 1: Experimental and computational as well as previously published data collected for the analyzed DRB1 allotypes.

DRB1 allele	HLA-DM susceptibility[$\mu M^{-1}min^{-1}$]$^{+}$	thermal stability [°C]$^{+0}$	k_{off} (150nM DM) [min^{-1}]$^{+}$	k_{off} (no DM) [min^{-1}]$^{+}$	G(MS1) [kJ/mol]§	G(MS2) [kJ/mol]§	p(MS1) [%]§	p(MS2) [%]§	p(MS3) [%]§	CLIP binding (%rank)#	CLIP binding (EL-score)#	OR(RA) [95%CI]*
*01:01	2.99±0.04E-02	82.1±1.2	5.12±0.48E-03	6.36±1.18E-04	6_{4}^{8}	9_{5}^{14}	8_{4}^{16}	2_{0}^{11}	90_{74}^{95}	1.65	0.64	1.38[1.28; 1.50]
*01:02	1.33±0.04E-01	87.3±0.0	2.14±0.40E-02	1.47±0.73E-03	8_{7}^{11}	13_{7}^{20}	4_{1}^{6}	1_{0}^{6}	96_{88}^{99}	1.38	0.71	0.93[0.66; 1.31]
*03:01	5.61±0.03E-01	73.6±0.1	1.03±0.05E-01	1.90±0.14E-02	2_{0}^{4}	21_{18}^{25}	30_{17}^{50}	0_{0}^{0}	70_{50}^{83}	4.94	0.23	0.59[0.54; 0.64]
*04:01	5.64±0.01E-01	64.2±0.6	8.71±0.09E-02	2.54±0.42E-03	2_{-1}^{4}	8_{5}^{14}	34_{17}^{56}	3_{0}^{5}	64_{38}^{82}	5.88	0.24	4.14[3.86; 4.44]
*04:04	3.74±0.01E-01	59.5±0.7	5.80±0.10E-02	1.93±0.16E-03	3_{1}^{5}	15_{12}^{20}	22_{13}^{40}	0_{0}^{1}	77_{59}^{87}	7.11	0.24	3.17[2.83; 3.54]
*07:01	4.30±0.23E-02	64.1±0.3	8.40±2.75E-03	1.95±0.44E-03	2_{0}^{4}	10_{5}^{15}	28_{17}^{47}	1_{0}^{7}	71_{47}^{82}	1.36	0.57	0.49[0.45; 0.54]
*08:01	5.20±0.11E-02	65.0±0.2	1.19±0.06E-02	4.13±1.87E-03	5_{3}^{7}	22_{13}^{25}	13_{6}^{24}	0_{0}^{0}	87_{76}^{94}	11.82	0.23	0.34[0.26; 0.44]
*08:02	2.71±0.01E-02	72.1±1.6	4.67±0.24E-03	6.03±1.97E-04	4_{1}^{5}	--	20_{13}^{40}	--	80_{60}^{87}	7.12	0.3	n.d.
*13:01	6.15±0.05E-02	73.5±0.8	1.40±0.26E-02	4.74±2.18E-03	8_{6}^{11}	24_{21}^{27}	5_{1}^{9}	0_{0}^{0}	95_{91}^{99}	11.61	0.22	0.28[0.24; 0.33]
*13:02	1.15±0.01E-01	80.9±2.8	2.61±0.27E-02	8.86±1.27E-03	9_{7}^{12}	10_{6}^{18}	3_{1}^{6}	2_{0}^{8}	95_{86}^{99}	4.93	0.3	0.29[0.23; 0.38]
*14:01	4.24±0.10E-01	69.6±0.1	6.73±1.03E-02	3.76±0.34E-03	3_{2}^{5}	9_{7}^{16}	21_{13}^{30}	2_{0}^{4}	77_{66}^{87}	4.17	0.34	0.46[0.36; 0.59]
*15:01	2.88±0.01E-01	74.2±0.6	5.66±0.15E-02	1.34±0.05E-02	1_{0}^{3}	9_{6}^{16}	36_{24}^{48}	2_{0}^{5}	62_{48}^{76}	11.89	0.06	0.57[0.53; 0.62]

+ Errors represent the standard deviation and are calculated from three independent experiments.
0 Thermal stability measurements are performed on the qPCR maschine MX 3005P (Stratagene).
§ Free energies and populations are shown with the 'lower-/upper-case' 1σ confidence interval.
# The binding score (EL and %rank) for the $CLIP_{103\text{-}116}$ peptide (PVSKMRMATPLLMQ) was obtained using the server NetMHCIIpan[1].
* The odds ratio (OR) for Rheumatoid Arthritis (RA) was extracted from the publication by Raychaudhuri et al.[2].

Supplementary Table 2: Thermal stability of DR1*01:01 and DR1*04:01 in the absence or presence of a ~100fold molar excess of CLIP peptide. Errors bars represent the standard deviation calculated from three (DR1*01:01) or four (DR1*04:01) independent experiments. Measurements were performed on the qPCR maschine StepOne Plus™ (Applied Biosystems).

DRB1 allele	thermal stability [°C]	
	no CLIP added	1mM CLIP added
*01:01	79.30 ± 1.32	82.34 ± 0.08
*04:01	63.25 ± 0.28	67.74 ± 0.54

Supplementary Table 3: Crystallographic data collection and refinement statistics.

	HLA-DRB1*04:01 CLIP	HLA-DRB1*07:01 CLIP	HLA-DRB1*01:02 fused CLIP
**Data collection**			
Beamline	BESSY 14.1	BESSY 14.1	BESSY 14.1
Wavelength (Å)	0.9184	0.9184	0.9184
Space group	$C222_1$	R3	$P2_1$
Cell dimensions			
a, b, c (Å)	96.9, 111.5, 212.5	134.1, 134.1, 72.2	57.7, 120.8, 68.2
α, β, γ (°)	90.0, 90.0, 90.0	90.0, 90.0, 120.0	90.0, 108.8, 90.0
Resolution (Å)*	43.81 – 2.09 (2.17 – 2.09)	45.24 – 2.10 (2.23 – 2.10)	46.60 – 1.76 (1.86 – 1.76)
R_{meas}*	15.9 (166.1)	7.6 (255.9)	9.9 (76.1)
$< I / \sigma(I) >$*	9.69 (1.16)	12.06 (0.81)	11.39 (1.94)
CC1/2*	0.996 (0.441)	0.999 (0.44)	99.8 (71.6)
Completeness* (%)*	99.0 (96.6)	98.6 (97.2)	98.5 (98.0)
Redundancy	4.6 (4.7)	4.0 (4.0)	3.8 (3.8)
**Refinement**			
Resolution (Å)	2.09	2.10	1.76
No. reflections	67077	27878	328677
R_{work} / R_{free} (%)	18.70 / 22.21	21.46 / 24.30	16.72 /20.33
No. atoms			
Protein	6430	3020	6309
Ligand	90	36	44
Water	610	125	907
Mean *B* factor ($Å^2$)	49.47	92.77	22.76
R.m.s deviations			
Bond lengths (Å)	0.005	0.014	0.009
Bond angles (°)	0.741	1.31	0.96
Mol/AU	2	1	2

* Data in highest resolution shell are indicated in parenthesis.

Supplementary Table 4: Root mean square deviation (RMSD) values of Cα atoms calculated for crystal structures of CLIP-bound DRB1*01:02 (PDB 7YX9), DRB1*04:01 (PDB (7YXB), and DRB1*07:01 (PDB 7Z0Q) in comparison to CLIP-bound DRB1*01:01 (PDB 3PDO[3]). RMSD values are shown for the entire pMHC complex, the α1β1-domains forming the peptide binding groove of the MHC protein, and for the core residues (P1-P9) of the CLIP peptide.

DRB1 allele	pMHC	MHC α1β1-domains	CLIP peptide (core residues)
*01:02	0.39 Å	0.22 Å	0.18 Å
*04:01	0.37 Å	0.45 Å	0.23 Å
*07:01	0.57 Å	0.44 Å	0.42 Å

Supplementary Table 5: Initial apparent on-rates for all analyzed allotypes at different HLA-DM concentrations.

DRB1	k_{on} (1µM DM) [mP.min^{-1}]	k_{on} (0.5µM DM) [mP.min^{-1}]	k_{on} (0.25µM DM) [mP.min^{-1}]	k_{on} (0.125µM DM) [mP.min^{-1}]	k_{on} (0.00625µM DM) [mP.min^{-1}]	k_{on} (0.003125µM DM) [mP.min^{-1}]	k_{on} (0. 0015625µM DM) [mP.min^{-1}]	k_{on} (no DM) [mP.min^{-1}]
*01:01	16.19±3.59	13.64±1.5	9.90±2.77	8.22±0.88	5.84±1.93	4.97±0.43	3.33±0.39	0.05±0.03
*01:02	30.02±1.04	25.45±2.77	19.12±1.2	15.73±1.45	9.97±1.81	8.32±1.13	1.91±0.48	0.86±0.6
*03:01	46.54±0.84	45.89±1.39	39.40±1.41	32.85±2.13	17.80±3.84	12.67±2.92	7.93±1.26	0.95±0.87
*04:01	47.13±1.43	46.04±1.54	35.40±2.42	28.57±1.25	18.43±2.67	13.69±1.9	5.34±2.35	0.75±0.63
*04:04	46.21±1.84	39.52±1.1	27.66±1.14	18.19±1.4	10.33±1.58	8.49±1.48	2.43±0.43	0.36±0.31
*07:01	17.36±2.71	16.37±3.15	13.25±2.94	10.43±2.4	5.34±2.24	3.84±1.04	1.28±0.65	0.18±0.04
*08:01	18.86±1.86	14.85±1.94	10.88±0.93	6.84±0.29	3.31±0.28	1.72±0.65	0.48±0.41	0.51±0.08
*08:02	14.27±0.96	12.74±1.09	9.88±0.98	7.76±1.27	2.92±0.72	2.80±0.76	0.63±0.41	0.22±0.13
*13:01	22.89±1.28	19.12±1.02	12.39±0.74	10.57±1.19	5.55±1.12	5.26±0.61	2.03±0.33	1.79±0.79
*13:02	31.06±0.81	22.68±0.78	18.28±0.52	17.92±1.48	12.78±0.44	9.46±0.65	4.43±1.44	1.46±0.46
*14:01	47.04±0.77	41.08±1.46	27.57±0.86	24.02±1.51	12.20±0.8	11.22±1.16	7.40±0.18	1.95±0.2
*15:01	45.47±1.29	35.66±1.43	24.71±1.1	18.52±0.97	9.00±0.58	6.62±1.06	0.52±0.19	0.67±0.2

Supplementary Table 6. Overview of molecular dynamics (MD) simulations performed for the DRB1 allotypes in complex with CLIP peptide.

DRB1 allotype	number of simulations	aggregted simulation length [μs]
*01:01	241	157.3
*01:02	245	175.0
*03:01	248	173.3
*04:01	249	177.0
*04:04	248	156.6
*07:01	248	169.4
*08:01	250	168.0
*08:02	249	172.5
*13:01	247	166.5
*13:02	244	166.9
*14:01	248	176.8
*15:01	250	171.4

Supplementary Table 7: Results of ^{1}H-^{13}C-methyl-CPMG analysis for DR1*01:01 residues.

DRB1*01:01 α-chain residues	methyl groups showing dynamics* $(\Delta(R_2^{eff}, R_2^0) > 2s^{-1})$	DRB1*01:01 β-chain residues	methyl groups showing dynamics* $(\Delta(R_2^{eff}, R_2^0) > 2s^{-1})$
α6Val	n.d./no	β8Leu	n.d./n.d
α10Ala	(yes)	β11Leu	n.d./(yes)
α14Leu	n.d./n.d.	β24Val	no/no
α34Val	n.d./n.d.	β26Leu	no/no
α37Ala	no	β27Leu	n.d./n.d.
α42Val	n.d./no	β31Ile	n.d.
α45Leu	n.d./no	β38Val	n.d./no
α52Ala	n.d.	β44Val	n.d./n.d.
α56Ala	no	β49Ala	no
α59Ala	(yes)	β50Val	n.d./no
α60Leu	**yes**/**yes**	β53Leu	n.d./n.d.
α61Ala	no	β58Ala	no
α64Ala	no	β67Leu	n.d./n.d.
α65Val	n.d./(yes)	β68Leu	n.d./no
α68Ala	no	β73Ala	no
α70Leu	n.d./no	β74Ala	no
α85Val	n.d./no	β75Val	no/no
α89Val	n.d./no	β85Val	n.d./n.d.
α91Val	**yes**/no	β91Val	n.d./no
α92Leu	(yes)/no	β95Val	n.d./no
α97Val	no/no	β99Val	n.d./n.d.
α 99Leu	n.d./n.d.	β101Val	n.d./no
α104Val	n.d./n.d.	β109Leu	n.d./n.d.
α105Leu	no/no	β114Leu	n.d./n.d.
α116Val	**yes**/no	β115Leu	no/no
α117Val	**yes**/**yes**	β116Val	n.d./no
α119Val	**yes**/**yes**	β119Val	n.d./n.d
α122Leu	no/no	β127Ile	n.d
α128Val	n.d./n.d.	β129Val	no/no
α132Val	**yes**/no	β140Ala	n.d.
α136Val	n.d./n.d.	β142Val	n.d./no
α138Leu	n.d./n.d.	β143Val	n.d./no
α144Leu	n.d./**yes**	β147Leu	n.d./no
α151Leu	**yes**/no	β148Ile	n.d.
α154Leu	n.d./n.d.	β158Leu	n.d./ n.d
α160Val	no/no	β159Val	n.d./ n.d
α165Val	no/no	β161Leu	no/no
α170Leu	n.d./no	β164Val	n.d./ n.d
α174Leu	n.d./no	β170Val	n.d./no
α175Leu	n.d./n.d.	β175Val	n.d./ n.d
α182Ala	no	β180Val	n.d./no
α186Leu	no/no	β184Leu	no/no
		β186Val	n.d./no
		β190Ala	n.d
		β195Ala	no

*n.d. means not determined, as either not assigned or overlapping peak
(yes): two-state exchange and no exchange models both fit data
yes: two-state exchange model selected

Supplementary Table 8: Values of the equilibrium dissociation constants (K_D) used in the double mutant cycle calculations of HLA-DR*01:01 mutants. Errors represent the standard deviation and are calculated from three independent experiments.

	K_D (nM)
DRB1*01:02	324.80±2.27
DRB1*01:01	414.47±14.83
DRB1*01:02(αN62A)	511.60±11.52
DRB1*01:01(αN62A)	1111.77±59.84
DRB1*01:02(βR71A)	507.70±1.15
DRB1*01:01(βR71A)	1357.33±17.60

Supplementary Table 9: Interaction free energies of the double mutant cycles (all units in kJ/mol). Errors represent the standard deviation and are calculated from three independent experiments.

	αN62A	βR71A
ΔG1	0.60±0.09	0.60±0.09
ΔG2	1.13±0.06	1.11±0.02
ΔG12	3.03±0.14	3.54±0.02
ΔΔG	1.30±0.14	1.83±0.02

Supplementary references

1. Reynisson, B. et al. Improved Prediction of MHC II Antigen Presentation through Integration and Motif Deconvolution of Mass Spectrometry MHC Eluted Ligand Data. *J Proteome Res* **19**, 2304-2315 (2020).
2. Raychaudhuri, S. et al. Five amino acids in three HLA proteins explain most of the association between MHC and seropositive rheumatoid arthritis. *Nat Genet* **44**, 291-6 (2012).
3. Gunther, S. et al. Bidirectional binding of invariant chain peptides to an MHC class II molecule. *Proc Natl Acad Sci U S A* **107**, 22219-24 (2010).
