# Supplementary material for: MHC-II dynamics are maintained in HLA-DR allotypes to ensure catalyzed peptide exchange
Source: Nat Chem Biol. 2023 May 4;19(10):1196–204. doi: 10.1038/s41589-023-01316-3 (PMC10522485; doi:10.1038/s41589-023-01316-3)
Supplement: Supplementary file 2 — Reporting Summary [file 41589_2023_1316_MOESM2_ESM.pdf]

nature portfolio

Corresponding author(s): Christian Freund, Frank Noe

Last updated by author(s): Mar 5, 2023

Reporting Summary

Nature Portfolio wishes to improve the reproducibility of the work that we publish. This form provides structure for consistency and transparency in reporting. For further information on Nature Portfolio policies, see our Editorial Policies and the Editorial Policy Checklist.

Statistics

For all statistical analyses, confirm that the following items are present in the figure legend, table legend, main text, or Methods section.

n/a	Confirmed	
☐	☒	The exact sample size (n) for each experimental group/condition, given as a discrete number and unit of measurement
☐	☒	A statement on whether measurements were taken from distinct samples or whether the same sample was measured repeatedly
☐	☒	The statistical test(s) used AND whether they are one- or two-sided<br>*Only common tests should be described solely by name; describe more complex techniques in the Methods section.*
☒	☐	A description of all covariates tested
☒	☐	A description of any assumptions or corrections, such as tests of normality and adjustment for multiple comparisons
☐	☒	A full description of the statistical parameters including central tendency (e.g. means) or other basic estimates (e.g. regression coefficient) AND variation (e.g. standard deviation) or associated estimates of uncertainty (e.g. confidence intervals)
☐	☒	For null hypothesis testing, the test statistic (e.g. F, t, r) with confidence intervals, effect sizes, degrees of freedom and P value noted<br>*Give P values as exact values whenever suitable.*
☒	☐	For Bayesian analysis, information on the choice of priors and Markov chain Monte Carlo settings
☒	☐	For hierarchical and complex designs, identification of the appropriate level for tests and full reporting of outcomes
☒	☐	Estimates of effect sizes (e.g. Cohen's d, Pearson's r), indicating how they were calculated

Our web collection on statistics for biologists contains articles on many of the points above.

Software and code

Policy information about availability of computer code

Data collection	TopSpin3.2 (Bruker), VICTOR3V (PerkinElmer), XDSAPP suite
Data analysis	CcpNMR Analysis 2.4.2, NESSY, GraphPad Prism version 9.3.1, Inkscape 0.91 and 1.1, PyMol version 1.8.0.0, Phaser, Phenix, MOLPROBITY, PROPKA31, HTMD, PDB2PQR, PyEMMA, PySFD

For manuscripts utilizing custom algorithms or software that are central to the research but not yet described in published literature, software must be made available to editors and reviewers. We strongly encourage code deposition in a community repository (e.g. GitHub). See the Nature Portfolio guidelines for submitting code & software for further information.

Data

Policy information about availability of data

All manuscripts must include a data availability statement. This statement should provide the following information, where applicable:

- Accession codes, unique identifiers, or web links for publicly available datasets
- A description of any restrictions on data availability
- For clinical datasets or third party data, please ensure that the statement adheres to our policy

Crystal structures are deposited to the PDB for DRB1*01:02 (PDB entry 7YX9), DRB1*07:01 (PDB entry 7Z0Q), DRB1*04:01 (PDB entry 7YXB). Other data are available from the corresponding author upon reasonable request.

Human research participants

Policy information about studies involving human research participants and Sex and Gender in Research.

Reporting on sex and gender	N/A
Population characteristics	N/A
Recruitment	N/A
Ethics oversight	N/A

Note that full information on the approval of the study protocol must also be provided in the manuscript.

Field-specific reporting

Please select the one below that is the best fit for your research. If you are not sure, read the appropriate sections before making your selection.

☒ Life sciences ☐ Behavioural & social sciences ☐ Ecological, evolutionary & environmental sciences

For a reference copy of the document with all sections, see nature.com/documents/nr-reporting-summary-flat.pdf

Life sciences study design

All studies must disclose on these points even when the disclosure is negative.

Sample size	Biochemical data were retrieved from n=3 independent experiments, which corresponds to the field's standard. Sample size calculation was not performed.
Data exclusions	No data was excluded.
Replication	For all biochemical assays numbers of replicates were at least three independent experiments with three technical replicates each, as stated in the respective figure legends.
Randomization	Not applicable, as no group allocation for case control studies was performed.
Blinding	Not applicable, as no group allocation for case control studies was performed.

Reporting for specific materials, systems and methods

We require information from authors about some types of materials, experimental systems and methods used in many studies. Here, indicate whether each material, system or method listed is relevant to your study. If you are not sure if a list item applies to your research, read the appropriate section before selecting a response.

Materials & experimental systems

n/a	Involved in the study	
☐	☒	Antibodies
☐	☒	Eukaryotic cell lines
☒	☐	Palaeontology and archaeology
☒	☐	Animals and other organisms
☒	☐	Clinical data
☒	☐	Dual use research of concern

Methods

n/a	Involved in the study	
☒	☐	ChIP-seq
☒	☐	Flow cytometry
☒	☐	MRI-based neuroimaging

Antibodies

Antibodies used	Antibody L243 (L243 HB-55™, ATCC)
Validation	Validation is provided by the vendor (ATCC) and the L243 antibody is standard in the field for binding and detection of MHCII proteins.

Eukaryotic cell lines

Policy information about cell lines and Sex and Gender in Research

Cell line source(s)	Sf9 insect cells were purchased from ThermoFisher.
Authentication	No further authentication applicable.
Mycoplasma contamination	Cell lines are routinely tested for mycoplasma contamination in our lab and cells used were negative.
Commonly misidentified lines (See ICLAC register)	No misidentified lines were used.
